# Experimental Confirmation of a Predicted Porous Hydrogen‐Bonded Organic Framework

**DOI:** 10.1002/anie.202303167

**Published:** 2023-05-03

**Authors:** Caitlin E. Shields, Xue Wang, Thomas Fellowes, Rob Clowes, Linjiang Chen, Graeme M. Day, Anna G. Slater, John W. Ward, Marc A. Little, Andrew I. Cooper

**Affiliations:** ^1^ Materials Innovation Factory and Department of Chemistry University of Liverpool 51 Oxford Street Liverpool L7 3NY UK; ^2^ Leverhulme Research Centre for Functional Materials Design University of Liverpool 51 Oxford Street Liverpool L7 3NY UK; ^3^ School of Chemistry and School of Computer Sciences University of Birmingham Edgbaston Birmingham B15 2TT UK; ^4^ Computational Systems Chemistry, School of Chemistry University of Southampton B27, East Highfield Campus, University Road Southampton SO17 1BJ UK

**Keywords:** Crystal Engineering, Crystal Structure Prediction, Hydrogen-Bonded Organic Frameworks, Porous Materials

## Abstract

Hydrogen‐bonded organic frameworks (HOFs) with low densities and high porosities are rare and challenging to design because most molecules have a strong energetic preference for close packing. Crystal structure prediction (CSP) can rank the crystal packings available to an organic molecule based on their relative lattice energies. This has become a powerful tool for the *a priori* design of porous molecular crystals. Previously, we combined CSP with structure‐property predictions to generate energy‐structure‐function (ESF) maps for a series of triptycene‐based molecules with quinoxaline groups. From these ESF maps, triptycene trisquinoxalinedione (TH5) was predicted to form a previously unknown low‐energy HOF (TH5‐A) with a remarkably low density of 0.374 g cm^−3^ and three‐dimensional (3D) pores. Here, we demonstrate the reliability of those ESF maps by discovering this TH5‐A polymorph experimentally. This material has a high accessible surface area of 3,284 m^2^ g^−1^, as measured by nitrogen adsorption, making it one of the most porous HOFs reported to date.

## Graphical abstract

A hydrogen‐bonded framework that was predicted previously to have 3D porosity and a remarkably low density of 0.37 g cm^−3^ has been discovered experimentally. The structure of this framework matches the original prediction precisely and it has an accessible surface area of 3,284 m^2^ g^−1^, as measured by nitrogen adsorption, making it one of the most porous hydrogen‐bonded frameworks synthesized to date.

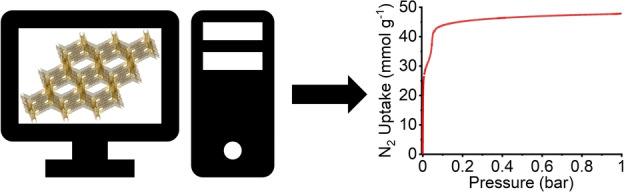

## Introduction

The design of new functional materials requires innovative design strategies.^[1]^ A well‐established approach to designing porous materials exploits strong, directional coordination clusters or covalent bonds to direct the packing of molecules in the solid state. This strategy has produced a wide variety of porous metal‐organic frameworks (MOFs)^[2]^ and covalent‐organic frameworks (COFs),^[3]^ mostly because of the structural predictability and stability of these framework bonding approaches.^[4]^ By contrast, structures composed of discrete molecules do not follow such reliable assembly patterns.^[5]^ This is because of the many weak, competing intermolecular interactions that dictate crystal packing, rather than a single, highly directional bonding motif.[^6^, ^7^]

Porous molecular crystals exhibit certain desirable properties that are rare in extended frameworks, such as solution processability.[^8^, ^9^] However, permanent porosity in molecular crystals is uncommon due to the propensity for molecules to pack closely in the solid state. The lack of strongly directional intermolecular bonding groups in many crystals can result in multiple competing polymorphs, each with different physical properties and separated in energy by only a few kilojoules per mole.^[10]^ The relative stability of these polymorphs is influenced by small changes to molecular structure; this means that even crystal engineering strategies, such as the introduction of directional hydrogen‐bonding moieties,^[11]^ rarely exert total control over crystallization.^[7]^

An alternative strategy to guide the discovery of porous molecular crystals, and crystalline organic materials more generally, is crystal structure prediction (CSP). In essence, CSP is a more quantified analogue of the reticular design concepts applied to extended frameworks,^[4]^ and it offers control over material function by predicting how candidate molecules will assemble in the solid state.[^12^, ^13^] Recently, we demonstrated how CSP could be combined with property prediction to generate energy‐structure‐function (ESF) maps.^[14]^ ESF maps enable the identification of experimentally accessible structures with desirable functions.^[15]^ The ability to screen candidate molecules *a priori* and focus experimental efforts is invaluable for materials with elaborate syntheses, such as many HOFs.[^16^, ^17^, ^18^, ^19^] In many cases, the timescale for ESF map computation is now much shorter than the timescale for synthesis, particularly for HOF building blocks that often have low solubilities and require significant synthetic route development.

HOFs are a class of porous crystalline material, typically constructed by functionalizing a rigid scaffold molecule with directional hydrogen bonding moieties.[^16^, ^17^, ^18^, ^19^] The intermolecular interaction of these hydrogen‐bonding groups frustrates the close packing of molecules in the solid state to generate porous scaffolds. However, other factors, such as interpenetration isomerism, can increase crystal density and affect the porosity of HOFs.[^20^, ^21^] While HOFs have become more numerous recently, they have yet to compete with frameworks such as MOFs and COFs in terms of surface areas and pore volume, even though they could have advantages, such as responsive or adaptive pore structures. There have been very few reports of permanently porous HOFs with a Brunauer–Emmett–Teller surface area (*SA*_BET_) greater than 2,000 m^2^ g^−1^,[^14^, ^22^, ^23^, ^24^, ^25^] mostly because of the difficulty generating hydrogen‐bond scaffolds with sufficiently low densities and desolvating such fragile materials.^[19]^ By contrast, such surface areas are now readily achievable in MOFs and COFs. The use of ESF maps to address this challenge was demonstrated by the discovery of the previously unknown γ‐polymorph of triptycene trisbenzimidazolone (T2), which had been reported by Mastalerz et al. to form the α‐polymorph with a *SA*_BET_=2,796 m^2^ g^−1^.^[22]^ ESF maps coupled with experimental searches led to the discovery of T2‐γ, which has the lowest density (0.412 g cm^−3^) and the highest *SA*_BET_ (3,425 m^2^ g^−1^) found for a HOF to date.

**Figure 1:**
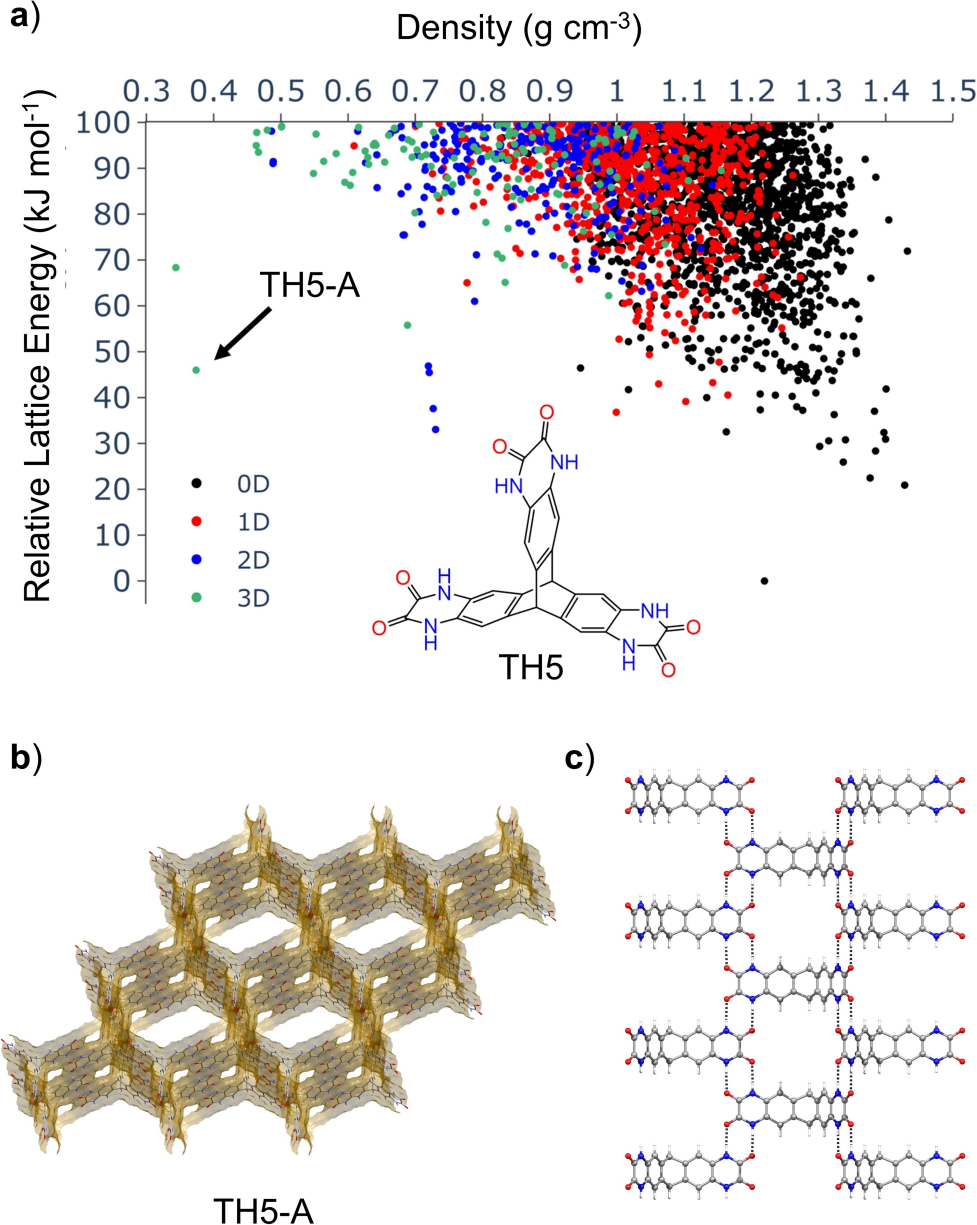
a) CSP landscape for TH5, showing dimensionality of the predicted pore channels, as reported in ref. [27]. The arrow indicates the polymorph TH5‐A at a predicted density of 0.374 g cm^−3^. Inset shows the chemical structure of TH5. b) Crystal packing in TH5‐A showing the contact surface using a 1.2 Å probe in Mercury.^[26]^ c) Hydrogen‐bonding motifs in TH5‐A.

More recently, we used CSP to assess an array of candidate molecules to investigate the effect of the position and the number of hydrogen bonding groups on HOF polymorphism.^[27]^ Included in this array was TH5 (Figure 1a), which comprises a triptycene core functionalized with three quinoxalinedione groups that interact to form a 3D hydrogen‐bonded network. The energy landscape of TH5 showed pronounced spikes corresponding to stable, low‐density polymorphs (Figure 1a). One of these polymorphs, TH5‐A (Figures 1b and 1c), was predicted to have an ultra‐low density of 0.374 g cm^−3^ and high surface area (predicted *SA*_BET_=4,265 m^2^ g^−1^). If the desolvated form of TH5‐A could be accessed, it would represent one of the most porous HOFs reported to date. TH5 has been synthesized previously,^[28]^ but this predicted crystal form, TH5‐A, was not observed, and these CSP predictions prompted us to reevaluate this molecule. Here, we report the experimental crystallization and activation of TH5‐A, referred to here as TH5α to denote the experimental structure, rather than the predicted form. We used supercritical CO_2_ (scCO_2_) drying as a gentle method for removing the solvent molecules from pores to retain the porosity.[^29^, ^30^, ^31^, ^32^]

## Results and Discussion

We synthesized TH5 using a four‐step procedure from triptycene (see Supporting Information, Section 1.7 and Scheme S1), following reported literature methods that avoid potentially explosive nitro‐containing intermediates.[^28^, ^33^, ^34^] The first step was a six‐fold bromination of triptycene using iron filings and bromine to afford 2,3,6,7,14,15‐hexabromotriptycene in 79 % yield. Next, 2,3,6,7,14,15‐hexabromotriptycene was reacted with benzophenone imine in a Pd^II^‐catalyzed cross‐coupling reaction to form intermediate **2** in 70 % yield. Finally, treatment of **2** with 2 M HCl (aq) afforded 2,3,6,7,14,15‐hexaaminotriptycene hexachloride salt (**3**) in 91 % yield, which was then reacted with diethyl oxalate to afford TH5 in 89 % yield. Solution ^1^H and ^13^C NMR spectra of TH5 and the reaction intermediates are consistent with the literature‐reported values[^28^, ^33^, ^34^] and indicate the clean formation of the desired product (Figures S1–8).

While CSP can determine crystal structures with low relative lattice energies and promising functions, it is computationally expensive to predict solvent templating effects.^[35]^ As such, CSP can suggest that a target phase might exist, but it does not tell us, in an affordable way, which solvents might access this phase. To address this, we screened the crystallization of TH5 experimentally using a range of conditions, searching for the predicted phase, TH5‐A. Initially, we screened the solubility of TH5 in 15 common organic solvents (Table S1). From this solubility screen, we found six ‘good’ solvents for TH5: *N,N*‐dimethylformamide (DMF), *N,N*‐diethylformamide (DEF), *N,N*‐dibutylformamide (DBF), *N,N*‐dimethylacetamide (DMAc), *N*‐methyl‐2‐pyrrolidone (NMP), and dimethyl sulfoxide (DMSO), all of which could dissolve five milligrams of TH5 in one milliliter of solvent at room temperature. We then used the remaining nine solvents that dissolved TH5 poorly under the same conditions as antisolvents; that is, tetrahydrofuran (THF), diethyl ether (Et_2_O), methanol (MeOH), ethanol (EtOH), chloroform (CHCl_3_), acetone, 2‐propanol (IPA), 1,4‐dioxane, and ethyl acetate. Different combinations of these good and bad solvents were then used for vial‐in‐vial vapour diffusion crystallizations, resulting in a total of 35 different crystallization conditions being screened (Table S2). Seven of these 35 crystallizations yielded crystals suitable for analysis by single‐crystal X‐ray diffraction (SCXRD) (Table S3).

**Figure 2:**
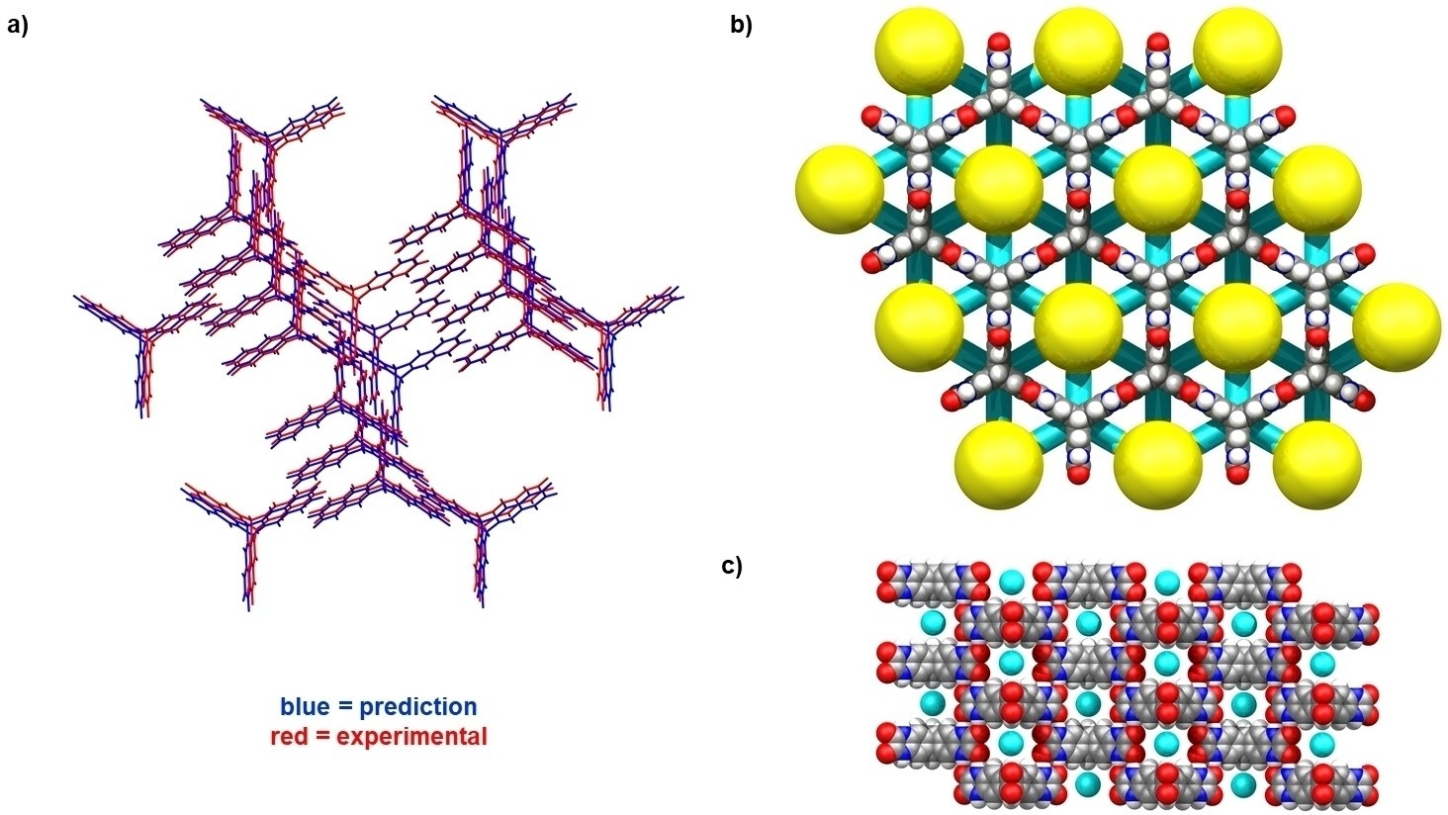
a) Crystal packing similarity search performed in Mercury^[26]^ between the CSP structure, TH5‐A (blue)^[27]^ and TH5α‐acetone (red). The search yielded an RMSD of 0.399 Å for a 20‐molecule comparison. b, c) Crystal packing in TH5α‐Acetone showing hexagonal pores (yellow) running along the crystallographic *c*‐axis and interconnecting channels (turquoise) running along the crystallographic *a* and *b*‐axes. Solvent molecules are omitted for clarity.

Slow diffusion of CHCl_3_ into a saturated solution of TH5 in DMF yielded orange, needle‐shaped crystals (Figure S9). SCXRD analysis revealed that the experimental crystal structure (TH5α‐DMF‐CHCl_3_) crystallized in hexagonal space group *P*6_3_/*mmc*. We found that exchanging the crystallization solvent in TH5α‐DMF‐CHCl_3_ with acetone to afford TH5α‐acetone improved the crystal stability and made single‐crystal selection more straightforward. We chose acetone because it is miscible with DMF and CHCl_3_, and TH5 is poorly soluble in acetone; we attribute the better single‐crystal stability to the latter. We recorded the single crystal structure of TH5α‐acetone after immersing the crystals of TH5α‐DMF‐CHCl_3_ in acetone for five days (see Supporting Information, Section 1.9 for full details).The SCXRD structure of TH5α‐acetone was comparable to TH5α‐DMF‐CHCl_3_ (*a*=21.158(3) Å, *c*=10.965(1) Å, *V*=4,251(1) Å^3^ at −173 °C for TH5α‐DMF‐CHCl_3_ *vs a*=21.3647(12) Å, *c*=11.0335(5) Å, *V*=4,361.5(4) Å at −73 °C for TH5α‐acetone). In the SCXRD structure of TH5α‐acetone, each TH5 molecule hydrogen bonds with six neighbouring TH5 molecules through a self‐complementary pair of short hydrogen bonds (r(N…O)=2.807(9) Å) to generate a 3D HOF of hexagonally packed TH5 molecules. Notably, the TH5α‐acetone crystal structure matches precisely with the predicted polymorph, TH5‐A, at a crystal framework density (ignoring solvent) of 0.374 g cm^−3^ (Figure 2a).

In the experimental TH5α‐acetone structure, 1.95 nm‐sized hexagonal‐shaped pores run along the crystallographic *c*‐axis (Figure 2b, yellow). In addition, ≈0.5 nm‐sized pores run orthogonally through the hexagonally shaped pores along the *a* and *b* unit cell axes (Figure 2b&c, turquoise), as predicted.^[24]^ As a result, TH5α‐acetone has 3D interconnected porosity, unlike other triptycene‐based molecules that have been reported to form hexagonally‐packed 1D channels, such as the trisbenzimidazolone T2‐γ,^[14]^ trisimidazole FDM‐15,^[36]^ and chalcogen‐bonded organic framework Trip3Sez.^[37]^ While other triptycene‐based HOFs with 3D pores have been reported, those hydrogen‐bonded networks have tended to interpenetrate.^[20]^ Hence, TH5α‐acetone is a rare example of a non‐interpenetrated triptycene‐based HOF that features interconnected 3D porosity.

We determined two other SCXRD structures of TH5, crystallized from DMF/THF with *Pbca* symmetry (TH5‐DMF‐THF) and DMSO/EtOH with *C*222_1_ symmetry (TH5‐DMSO‐EtOH) from the same crystallization study (Table S3, Figures S11–S14). However, TH5‐DMF‐CHCl_3_ and TH5‐DMSO‐EtOH feature solvent molecules hydrogen‐bonded to the TH5 molecules, preventing the formation of extended hydrogen‐bonded networks. In both cases, the solvent molecules affect how the TH5 molecules crystallize profoundly, and neither structure would have been predicted without the inclusion of multiple solvent molecules in the CSP search. Solvent interference with HOF formation through strong solvent interactions with the host molecule could be anticipated using CSP methods developed for solvate structure and stoichiometry prediction, which could predict whether stable solvate structures involve hydrogen bonding to solvent molecules. However, such methods are computationally demanding and have, to date, only been demonstrated on small model systems.^[38]^ Since solvent was not considered in our previous CSP study, neither of these solvated crystal structures matched any of the predicted landmark structures.

**Figure 3:**
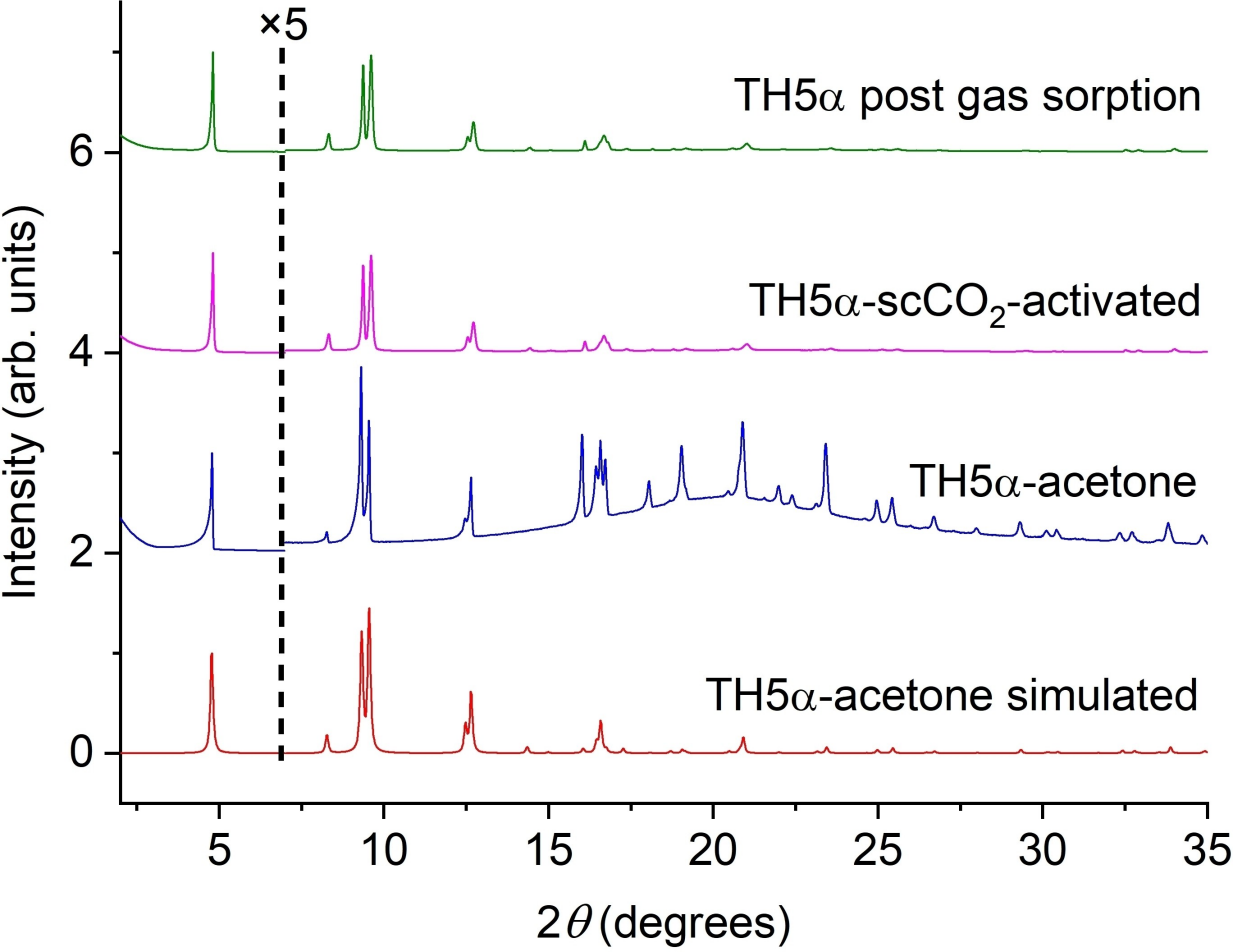
PXRD patterns for TH5α, showing simulated PXRD pattern from the SCXRD of TH5α‐acetone (red), experimental PXRD pattern TH5α‐acetone solvate recorded in the acetone solvent in a capillary (blue), TH5α‐scCO_2_‐activated (magenta), and TH5α recorded post gas sorption. Intensities (arb. units) are multiplied ×5 from 2*θ*=7 (from the vertical dashed line) for clarity.

The primary aim of this study was to find suitable crystallization conditions that afforded TH5‐A with a predicted density of 0.374 g cm^−3^, which would represent the lowest density molecular crystal structure reported in the Cambridge Structural Database to date.^[39]^ To investigate the solvent‐free stability of TH5α, we scaled up the crystallization (see Supporting Information, Section 1.9) and attempted bulk activation of the TH5α‐DMF‐CHCl_3_ material. Initially, we ran capillary powder X‐ray diffraction (PXRD) measurements of TH5α‐DMF‐CHCl_3_, which indicated that the material was phase pure and that the crystal structure was thermally stable up to at least 120 °C (Figure S15). However, due to the extremely low experimental skeleton density of the hydrogen‐bonded framework in TH5α‐DMF‐CHCl_3_, we anticipated that desolvation of the crystal pores might be challenging. Hence, to use a lower activation temperature, we exchanged the DMF and CHCl_3_ crystallization solvent in the crystal pores, initially with acetone and, subsequently, with *n*‐pentane. The PXRD pattern for the bulk TH5α‐acetone material also closely matched the simulated PXRD pattern from the TH5α‐acetone SCXRD structure (Figure 3).

We exchanged the acetone in the pores of TH5α‐acetone with *n*‐pentane to form TH5α‐pentane (see Supporting Information, Section 1.10 for full details). Activation was then attempted under vacuum at 25 °C, 55 °C, and 90 °C for TH5α‐acetone, and at 25 °C for TH5α‐pentane. However, despite the mild activation conditions, the removal of the solvent under vacuum led to a collapse of the structure and loss of crystallinity, as evidenced by PXRD (Figure S18). Furthermore, gas sorption analysis of the TH5α‐acetone activated at 25 °C showed a very low *SA*_BET_ of 112 m^2^ g^−1^, as calculated from the N_2_ sorption isotherm at 77 K (Figure S19–20). In future studies, the stability of activated HOFs could be explored computationally, in advance of designing conditions for activation, using recently‐developed methods for characterizing the energy landscapes of molecular crystals and quantifying the energy barriers to the collapse of predicted HOF structures. These methods have been used retrospectively to rationalize the stability of T2‐γ.^[40]^

We next attempted to activate TH5α‐DMF‐CHCl_3_ using a supercritical CO_2_ (scCO_2_) drying method. This technique is commonly used to desolvate low‐density MOFs,[^29^, ^30^] but has only recently been used to desolvate HOFs.[^31^, ^32^] We first exchanged the crystallization solvent with acetone because of the miscibility of acetone in CO_2_ and the apparent improved single‐crystal stability of TH5α‐acetone. The PXRD pattern of the scCO_2_‐activated material (TH5α‐scCO_2_‐activated) was an excellent match to the simulated PXRD pattern from TH5α‐acetone (Figure 3). Hence, unlike the acetone and *n*‐pentane exchanged materials after vacuum activation, the TH5α‐scCO_2_‐activated material appeared to maintain crystallinity after activation.

**Figure 4:**
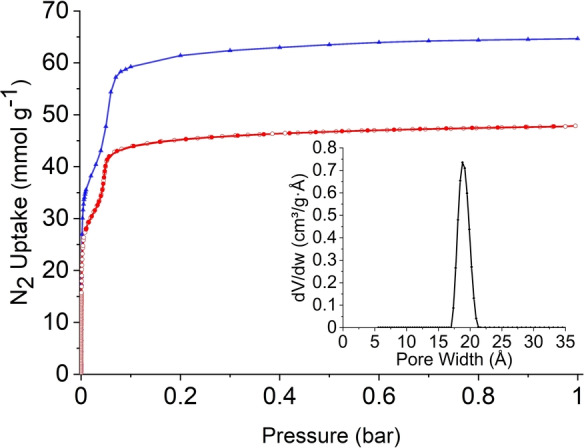
N_2_ sorption isotherms for TH5α‐scCO_2_‐activated recorded at 77 K (red, circles), and the predicted N_2_ adsorption isotherm for TH5‐A (blue, triangles); filled circles, adsorption experiment; unfilled circles, desorption experiment. The insert shows the pore size distribution plot for TH5α‐scCO_2_‐activated.

TH5α‐scCO_2_‐activated exhibited a Type IV(b) isotherm with a step at *P*/*P*_0_=0.04, indicative of capillary condensation within small mesopores (Figure 4). The experimental *SA*_BET_ was estimated to be 3,284 m^2^ g^−1^ (Figure S21), a large improvement on the vacuum‐activated TH5α‐acetone material and, to our knowledge, the second‐highest surface area reported for a HOF to date.[^14^, ^17^, ^41^] Furthermore, TH5α also has a large calculated pore width of 1.9 nm, in agreement with the predicted TH5‐A structure, and a total pore volume of 1.66 cm^3^ g^−1^. Although the predicted *SA*_BET_ for TH5‐A was estimated to be 4,265 m^2^ g^−1^ (see Supporting Information, Section 3 and Figures 4 and S22), thermogravimetric analysis (TGA) and NMR spectroscopy of the TH5α material after gas sorption (Figure S23–24) showed that strongly‐bound acetone (approximately 10 wt.%) remained in the pores. This remaining acetone solvent could partly account for the experimental surface area being lower than the predicted value. Increasing the degas temperature to 40 °C removed almost all acetone from the pores (Figure S25), but significantly reduced the *SA*_BET_ to 2,315 m^2^ g^−1^ (Figure S26–27). While there was no observable change in the PXRD pattern (Figure S28), these data indicate that full desolvation may lead to a partial collapse of the crystal structure. We note here that amorphous material may be relatively invisible to PXRD, particularly at low levels where there may be little evidence of an amorphous background.

## Conclusion

In summary, a computationally predicted HOF, TH5‐A,^[27]^ has been realized experimentally, offering further evidence that CSP is a powerful method for *a priori* functional materials design. Using a crystallization screen, we were able to identify conditions to access the predicted TH5‐A polymorph and confirm its crystal structure using SCXRD. Successful activation of TH5α was achieved using a scCO_2_ drying technique, which led to a large increase in porosity compared to conventional heat and vacuum activation methods.

While it was not possible to fully remove all solvent from the pores without partial collapse of the crystal structure, the experimental *SA*_BET_ of partially solvated TH5α was found to be 3,284 m^2^ g^−1^, making it, to our knowledge, the second most porous HOF to date based on this measure.[^14^, ^17^, ^41^] TH5α is also the first example of a highly porous HOF using quinoxaline hydrogen‐bonding functionality. Without computational methods to direct the synthetic efforts, TH5α may never have been discovered. We also emphasize that the two most porous HOFs discovered to date, T2‐γ and TH5α, were identified using ESF maps such that, in both cases, we knew what phase we were looking for, and why. This is a very different approach to open‐ended screening, where each new candidate's experimental phase represents a total unknown. This is particularly advantageous for materials, such as HOFs, where the growth of single crystals suitable for X‐ray diffraction can be challenging, but where PXRD for phase identification is typically more facile.

## Conflict of interest

The authors declare no conflict of interest.

1

## Supporting information

As a service to our authors and readers, this journal provides supporting information supplied by the authors. Such materials are peer reviewed and may be re‐organized for online delivery, but are not copy‐edited or typeset. Technical support issues arising from supporting information (other than missing files) should be addressed to the authors.

Supporting Information

Supporting Information

Supporting Information

Supporting Information

## Data Availability

The data that support the findings of this study are available in the supplementary material of this article.
