# Supplementary material for: Experimental Confirmation of a Predicted Porous Hydrogen‐Bonded Organic Framework
Source: Angew Chem Int Ed Engl. 2023 May 3;62(34):e202303167. doi: 10.1002/anie.202303167 (PMC10952618; doi:10.1002/anie.202303167)
Supplement: Supplementary file 1 — Supporting Information [file ANIE-62-0-s002.pdf]

Angewandte Chemie
Eine Zeitschrift der Gesellschaft Deutscher Chemiker

Supporting Information

Experimental Confirmation of a Predicted Porous Hydrogen-Bonded Organic Framework

C. E. Shields, X. Wang, T. Fellowes, R. Clowes, L. Chen, G. M. Day, A. G. Slater, J. W. Ward*, M. A. Little*, A. I. Cooper**

1. Supplementary Materials and Methods

1.1 Materials

All chemicals and solvents were purchased from Sigma Aldrich, TCI Europe and Acros Organics. Anhydrous solvents were purchased from Sigma-Aldrich, Acros Organics or Fisher Scientific. All chemicals were used without further purification. All gases for sorption analysis were supplied by BOC at a purity of ≥99.9%. Reactions were carried out under a N_2 atmosphere using standard Schlenk techniques.

1.2 NMR

NMR spectra were recorded on a Bruker Advance 400 NMR spectrometer at 400.13 MHz (^{1}H) and 100.61 MHz (^{13}C). ^{13}C spectra are ^{1}H decoupled.

1.3 Thermal Gravimetric Analysis (TGA)

TGA analysis was carried out using a TA Q5000 IR analyzer with an automated vertical overhead thermobalance. Samples were heated under nitrogen at a rate of 10 °C/min up to 1000 °C.

1.4 Single Crystal X-ray Diffraction (SCXRD)

SCXRD data sets were measured on a Rigaku AFC12K-007 HF rotating anode diffractometer (Mo-Kα radiation, λ = 0.71073 Å, Kappa 4-circle goniometer, HyPix-6000HE detector). Data reduction was performed using the CrysAlisPro software. Solvated single crystals were isolated from the crystallization solvent, immersed in a protective oil, mounted on a MiTeGen loop, and flash-cooled to 100 K under a dry N_2 gas flow. Structures were solved with SHELXT[1] and refined by full-matrix least-squares on $|F|^2$ by SHELXL,[2] interfaced through the programme OLEX2.[3] All non-H atoms were refined anisotropically, and all H-atoms were fixed in geometrically estimated positions and refined using the riding model. Where structures were found to contain disordered solvent molecules, the SQUEEZE[4] routine of PLATON[5] was used to remove scattering caused by disordered guests. For full SCXRD refinement details, see Section 3; for images of the crystal structure, see Figures S10–14.

1.5 Gas Sorption Analysis

Surface areas were measured by N_2 sorption at 77 K. Samples were degassed on the analysis port at room temperature under vacuum. Isotherm measurements were performed using a Micromeritics 3flex surface characterization analyzer. During analysis, the temperature was controlled using a *Cold-Edge Technologies* liquid He cryostat chiller unit. Apparent Brunauer–Emmett–Teller (BET) surface areas for TH5α were calculated using data in a relative pressure range chosen to fulfil best the criteria proposed by Rouquerol *et al*.[6]

1.6 Powder X-ray Diffraction (PXRD)

High-throughput PXRD patterns were collected in vertical transmission mode from loose powder samples held on Mylar film in aluminium well plates, using a Panalytical Empyrean equipped with a high throughput screening XYZ stage, X-ray focusing mirror and PIXcel detector with Cu-Kα radiation (λ = 1.541 Å). For solvated crystals and variable temperature experiments, samples were broken up with a glass pipette and loaded into 1 mm borosilicate glass capillaries that were spun during data collection to improve averaging. These PXRD patterns were collected in transmission mode on a

Panalytical Empyrean diffractometer equipped with a sample spinner, X-ray focusing mirror, and PIXcel 3-D detector in 1-D scanning mode with Cu-Kα radiation (λ = 1.541 Å). For non-ambient temperature PXRD measurements, the capillary temperature was controlled using an Oxford Cryosystems 700 Series Cryostream Plus.

1.7 Synthetic procedure

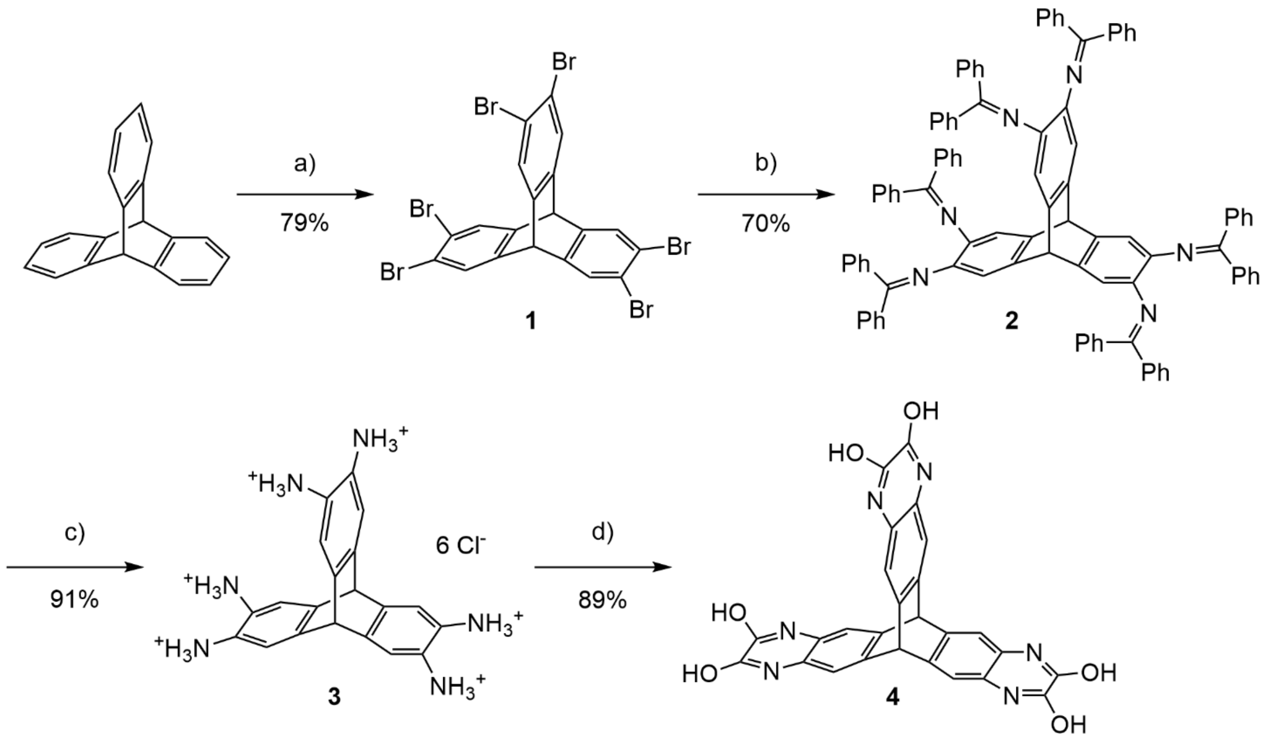

Scheme S1: Synthesis of TH5. Reagents and conditions: a) Br_2, Fe, $CHCl_3$, reflux, 1 hour; b) $(C_6H_5)_2C=NH$, $[Pd_2(dba)_3]$, *rac*-BINAP, sodium *t*-butoxide, toluene, 110 °C, overnight; c) 2M HCl, THF, 25 °C, 0.5 hours; d) diethyl oxalate, water, 100 °C, 20 hours.

Synthesis of 2,3,6,7,14,15-hexabromotriptycene (1): Triptycene (1.06 g, 4.18 mmol) was dissolved in $CHCl_3$ (80 mL) in a round-bottom flask. Iron filings (0.03 g, 0.54 mmol) were added, and the solution was stirred at 25 °C. Bromine (1.35 mL, 26.33 mmol) was added, and the solution was refluxed for one hour, during which time the initially reddish-brown solution turned reddish-orange. The flask was removed from heat, and upon cooling to room temperature, the reaction system was washed with saturated aqueous sodium thiosulfate solution until colourless. The organic layer was then separated and evaporated to dryness under reduced pressure. The resulting brown powder was dissolved in $CHCl_3$ (100 mL) and flushed through a pad of silica using additional $CHCl_3$ as eluent (100 mL). The filtrate was evaporated to dryness. The crude product was recrystallized from $CHCl_3$ to give the pure product as colourless, needle-like crystals (2.39 g, 79%). ^1H NMR ($CDCl_3$, 400 MHz): δ 7.54 (s, 6H), 5.16 (s, 2H) ppm. ^{13}C NMR ($CDCl_3$, 100 MHz): δ 143.96, 129.09, 121.78, 51.11 ppm. All values are consistent with literature-reported values.[7]

Synthesis of $N^2,N^3,N^6,N^7,N^{14},N^{15}$-Hexakis(diphenylmethylene)triptycene-2,3,6,7,14,15-hexamine (2): Tris(dibenzylideneacetone)dipalladium(0) (0.44 g, 0.46 mmol) and *rac*-BINAP (0.61 g, 0.97 mmol) were added to a 100 mL two-necked flask under a N_2 atmosphere. Anhydrous toluene (35 mL) was added, and the solution was refluxed for 30 minutes under N_2. The solution was cooled to room temperature, then benzophenone imine (2.5 mL, 14.82 mmol), **1** (1.32 g, 1.81 mmol), and sodium *t*-

butoxide (1.43 g, 14.82 mmol) were added, and the mixture was refluxed overnight. The resultant solution was cooled to room temperature, diluted with DCM (35 mL), filtered (Whatman®, 55 mm ø), and evaporated to dryness under reduced pressure. Recrystallization from ethyl acetate/EtOH afforded the product as a yellowish-orange solid (1.68 g, 70%). ^{1}H NMR (DMSO-d_6, 400 MHz): δ 7.49 (m, 12H), 7.37 (m, 24H), 7.20 (t, 12H), 6.72 (d, 12H), 6.28 (s, 6H), 4.49 (s, 2H) ppm. ^{13}C NMR (DMSO-d_6, 100 MHz): δ 166.61, 140.64, 140.04, 137.55, 136.70, 130.97, 129.20, 129.07, 128.64, 128.32, 116.44, 51.16 ppm. All values are consistent with literature-reported values.[8]

Synthesis of 2,3,6,7,14,15-Hexaaminotriptycene.6HCl (3): 2.0 M aqueous HCl solution (0.35 mL, 0.68 mmol) was added to a THF solution (5.0 mL) of **2** (100 mg, 0.08 mmol) and the mixture was stirred at room temperature for 30 minutes. The precipitate was isolated by filtration, washed twice with THF, ethyl acetate, and hexane (20 mL) sequentially, and dried under vacuum to give the hexaammoniumtriptycene hexachloride salt **3** as an off-white solid (39 mg, 91%). ^{1}H NMR (400 MHz, D_2O) δ 7.14 (s, 6H), 5.41 (s, 2H) ppm. ^{13}C NMR (100 MHz, D_2O) δ 141.59, 125.41, 117.05, 50.48 ppm. All values are consistent with literature-reported values.[9]

Synthesis of 2,3,6,7,12,13-Hexahydroxy-2,6,12-trihydrotripty[2,3-*d*:6,7-*d′*:12,13-*d″*]tripyrazyl (TH5): **3** (140 mg, 0.25 mmol) and diethyl oxalate (0.1 mL) were dissolved in water (6 mL) and vigorously stirred. After 20 hours, the reaction mixture was cooled to room temperature and the off-white solid was collected on a Büchner funnel and washed with water (3 x 4 mL). After being dried in vacuo, TH5 remained as a pale brown solid (113 mg, 89%): ^{1}H NMR (400 MHz, DMSO-d_6) δ 11.92 (s, 6H), 7.18 (s, 6H), 5.69 (s, 2H) ppm. ^{13}C NMR (100 MHz, DMSO-d_6) δ 155.74, 140.43, 122.79, 111.19, 50.55 ppm. HRMS (ESI +ve): [M+Na$^+$] calcd. for $C_{26}H_{14}N_6O_6Na^+$, 529.0867; found, 529.0858. All values are consistent with literature-reported values.[9]

1.8 Solubility Testing and Screening of Crystallization Conditions

Table S1: Solvents used in the experimental crystallization screen of TH5. Solvents that dissolved ≥ 5 mg of TH5 in 1 mL at room temperature are marked with an asterisk (*). The solvents that dissolved < 5 mg of TH5 in 1 mL at room temperature are marked with a cross ($^+$).

**N,N*-dimethyl formamide (DMF)	**N,N*-diethyl formamide (DEF)	* *N,N*-dibutyl formamide (DBF)	**N,N*-Dimethyl acetamide (DMAc)	**N*-methyl-2-pyrrolidone (NMP)
*Dimethyl sulfoxide (DMSO)	$^+$Tetrahydrofuran (THF)	$^+$ Diethyl ether (Et_2O)	$^+$ Methanol (MeOH)	$^+$ Ethanol (EtOH)
$^+$ $CHCl_3$ (chloroform)	$^+$ Acetone	$^+$ 2-propanol (IPA)	$^+$ 1,4-Dioxane	$^+$ Ethyl acetate

Fifteen solvents were screened for the TH5 crystallization experiments (Table S1). To determine the solubility of the target compound, 1 mL of each solvent was added to a vial containing 5 mg TH5. The vials were then agitated at room temperature. The solvents which fully dissolved TH5 at this concentration (DMF, DEF, DBF, DMAc, NMP and DMSO) were chosen as good solvents for the crystallization screen. The remaining solvents in which TH5 was poorly soluble under the same conditions were used as antisolvents. Good solvents were then used to prepare 10 mg mL^{-1} stock solutions of TH5. Crystals of TH5 were grown using the vial-in-vial vapour diffusion technique. Sample vials containing the TH5 stock were capped with a pierced plastic lid and placed inside a larger sample

vial containing 4 mL antisolvent. The larger vials were capped and left at room temperature. Crystallizations using THF, Et_2O, MeOH, and EtOH as the antisolvent were trialled first. From these initial experiments, only the DMF/THF and DMSO/EtOH combinations yielded crystals within two weeks. Additional experiments were then set up using DMF and DMSO as 'good' solvents, and $CHCl_3$, acetone, IPA, 1.4-dioxane, and ethyl acetate as 'bad' solvents. The results of the full crystallization screen are shown in Table S2.

Table S2: Results of crystallization screening. Blank boxes (-) indicate there was no precipitate formation over the timescale of the screening (two weeks). Grey boxes show solvent combinations that were not trialled.

	***DEF**	***DBF**	***DMAc**	***NMP**	***DMSO**	***DMF**
**$^+$THF**	-	powder	powder	powder	-	**crystals**
**$^+Et_2O$**	powder	powder	powder	powder	powder	powder
**$^+$MeOH**	powder	powder	-	powder	-	powder
**$^+$EtOH**	-	powder	powder	powder	**crystals**	powder
**$^+CHCl_3$**					crystals	**crystals**
**$^+$Acetone**					crystals	**crystals**
**$^+$IPA**					-	**crystals**
**$^+$1,4-Dioxane**					crystals	**crystals**
**$^+$Ethyl acetate**					crystals	**crystals**

From the solvent combinations screened, seven experiments yielded crystals of sufficient quality to obtain single-crystal XRD structures, which are highlighted in bold in Table S2. Single crystals of TH5 were also prepared by slow evaporation of a saturated solution of TH5 in NMP. The data for all successful crystallization experiments can be found in Table S3.

1.9 Scale-up of TH5α-acetone

As-synthesized TH5 (50 mg, 0.1 mmol) was dissolved in DMF (20 mL) with stirring. The solution was filtered through filter paper (Whatman®, 55 mm ø) into a 40 mL vial to remove any insoluble material. The vial was then closed with a septum pierced several times with a needle and placed inside a duran bottle containing $CHCl_3$ (20 mL). The bottle was sealed and left at room temperature. Vapour diffusion of $CHCl_3$ into the TH5 solution was carried out for several days until the vial was full. After one week, brown, needle-shaped crystals formed around the rim of the vial. The solvent was removed from the vial, leaving just enough to cover the crystals, and replaced with acetone. The vial was left to stand for half an hour, and then the solvent was exchanged with fresh acetone. This procedure was repeated several more times, and then the crystals were left to acetone exchange for one week, with the solvent being replaced with fresh acetone approximately every 24 hours.

1.10 Activation of TH5α-acetone

Two methods were trialled to remove solvent molecules from the TH5α HOF; heat/vacuum activation and supercritical CO_2 ($scCO_2$) drying. For heat/vacuum activation, crystals were exchanged with

acetone following the above procedure. One batch of crystals was then further exchanged with *n*-pentane. It should be noted that even after exchanging with pentane for two weeks, ~20 wt% of acetone was still present in the framework, as detected by ^{1}H NMR (Figure S17). TH5α-acetone crystals were then heated to 25 °C, 55 °C, and 90 °C under vacuum, and TH5α-pentane crystals were heated to 25 °C under vacuum. As determined by PXRD, all attempts resulted in the framework collapsing (Figure S18). For activation by scCO_2, crystals of TH5α-acetone were transferred to porous pots and left to soak in acetone for one week. The pots were then transferred to a sample holder filled with acetone and loaded into the supercritical drying chamber. The chamber was filled with liquid CO_2, and then drained for five minutes to remove acetone from the sample holder. This process was repeated immediately, then approximately every hour for six hours. The solvent level in the chamber was kept above the level of the sample holder at all times to prevent the crystals from drying out. After six hours of venting and soaking, the temperature in the chamber was raised to 40 °C, which raised the pressure to above the critical point of CO_2 (>1000 psi). The sample was held at the critical point for 15 minutes, and then the chamber was slowly vented over one and a half hours. Once the chamber was at atmospheric pressure, the sample was removed and transferred immediately to the gas sorption instrument.

2. Computational Methods

Crystal structure prediction (CSP). Crystal structure prediction for TH5 was reported previously.[10] These calculations used a rigid-molecule approach, where the DFT-optimized (B3LYP/6-311G(d,p)) molecular geometry was fixed throughout CSP and trial crystal structures were generated in 23 common space groups with one molecule in the asymmetric unit using the Global Lattice Energy Explorer software.[11] This approach generates trial crystal structures using a low-discrepancy, quasi-random sampling of molecular positions, molecular orientations and unit cell parameters. Trial crystal structures were energy minimized using an anisotropic atom-atom force field using the DMACRYS[12] software and structure generation was performed until 5000 structures had been successfully energy minimized in each space group (115,000 energy minimized crystal structures in total). Duplicate crystal structures were removed by comparing simulated powder X-ray diffraction patterns, followed by structure overlays using the COMPACK algorithm.[13]

Gas adsorption. Gas adsorption simulations were performed using grand-canonical Monte Carlo (GCMC) simulations involving a 50,000-cycle equilibration period and a 50,000-cycle production run. We performed these simulations using the CSP-predicted TH5-A structure reported in a previous study.[10] The input of a GCMC simulation includes the temperature and chemical potential of the gas molecules in the reservoir, and the output of the simulation is the average number of adsorbed molecules. This is analogous to an adsorption experiment, in which the temperature and bulk pressure of a gas are specified, and the corresponding uptake is measured. The chemical potential, as used in GCMC simulations, can be related to the gas-phase pressure, as specified in experiments, by an equation of state. The Peng–Robinson equation was used here. The trial MC moves included insertion, deletion, translation, rotation, and reinsertion; these moves were randomly attempted with equal probabilities. GCMC simulations were performed using the RASPA package.[14]

The adsorbent–adsorbate and adsorbate–adsorbate intermolecular interactions were modelled using Lennard–Jones (LJ) and Coulomb potentials. A cut-off radius of 12.0 Å was used for all LJ interactions (simple truncation), while all Coulomb interactions were computed using the Ewald summation technique with a relative precision of 10^{-6}. The LJ parameters for the adsorbent structures were assigned based on the DREIDING force field.[15] The Lorentz–Berthelot combining rules were used to calculate the LJ cross-parameters. The N_2 molecule was described by the TraPPE force field.[16]

To obtain partial atomic charges of TH5, an isolated TH5 molecule in the gas phase was used for calculations, performed at the B3LYP/6-31+G(d,p) level of theory with the Gaussian 09 software.[17] The Merz–Kollman scheme was used to fit partial atomic charges to the first- principles electrostatic potential of the molecule, with the vdW exclusion radii of the H, C, N, and O atoms being 1.10, 1.70, 1.55, and 1.52 Å, respectively.

3. Supplementary Data

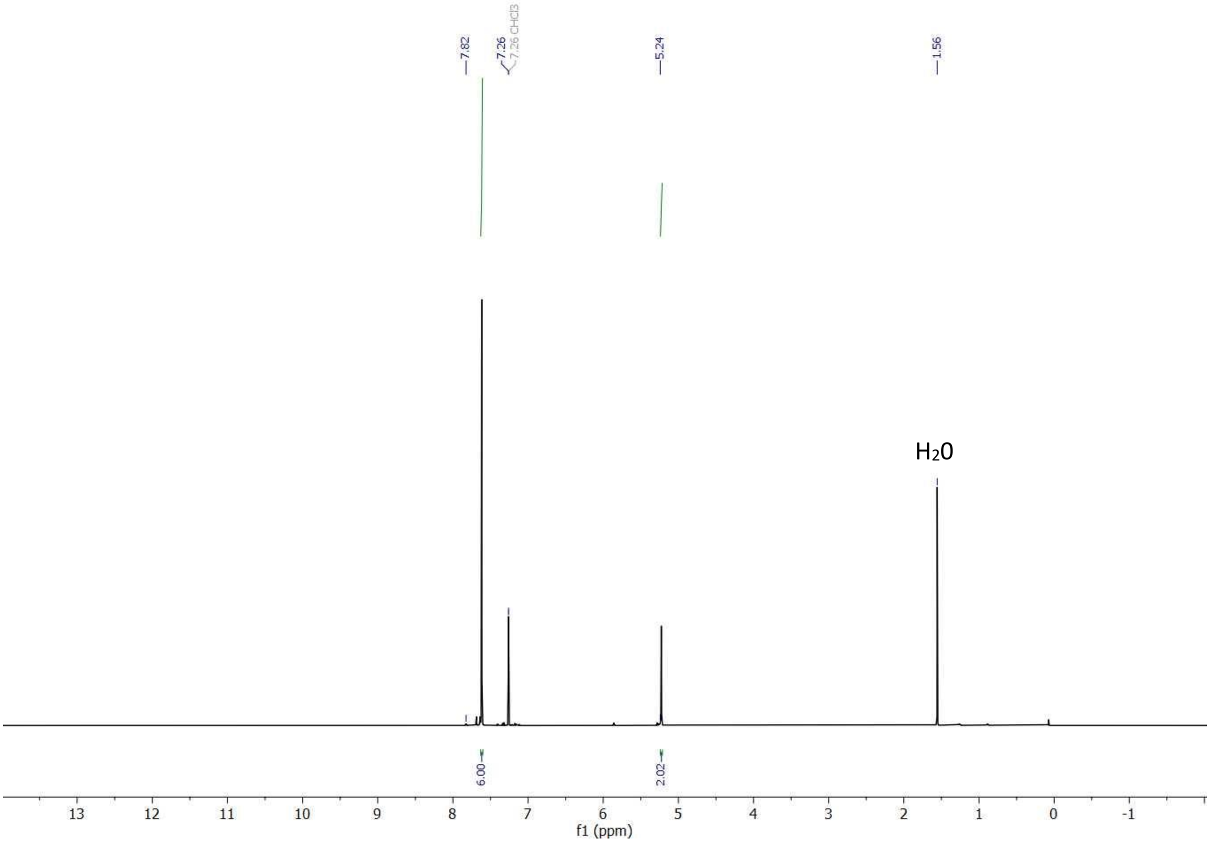

Figure S1: ^{1}H NMR spectrum ($CDCl_3$, 400 MHz) of compound **1**.

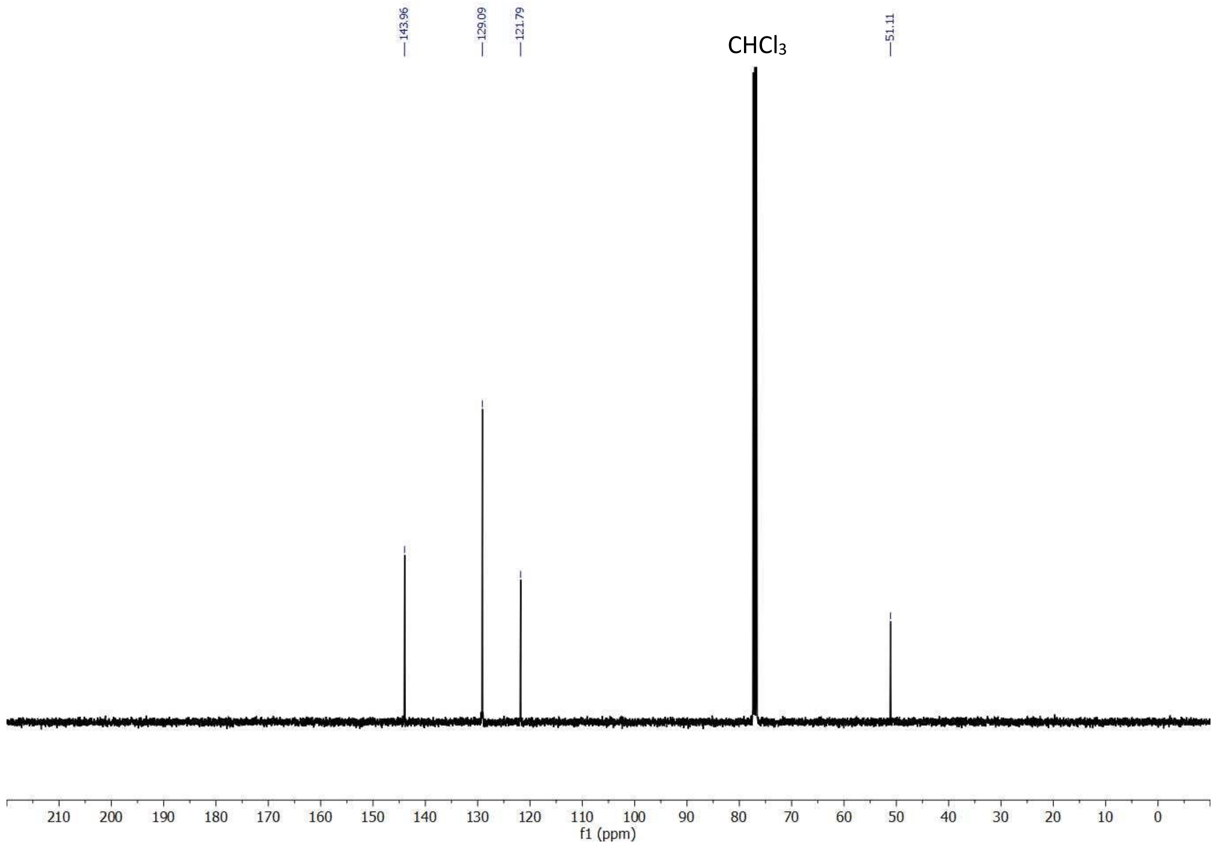

Figure S2: ^{13}C NMR spectrum ($CDCl_3$, 100 MHz) of compound **1**.

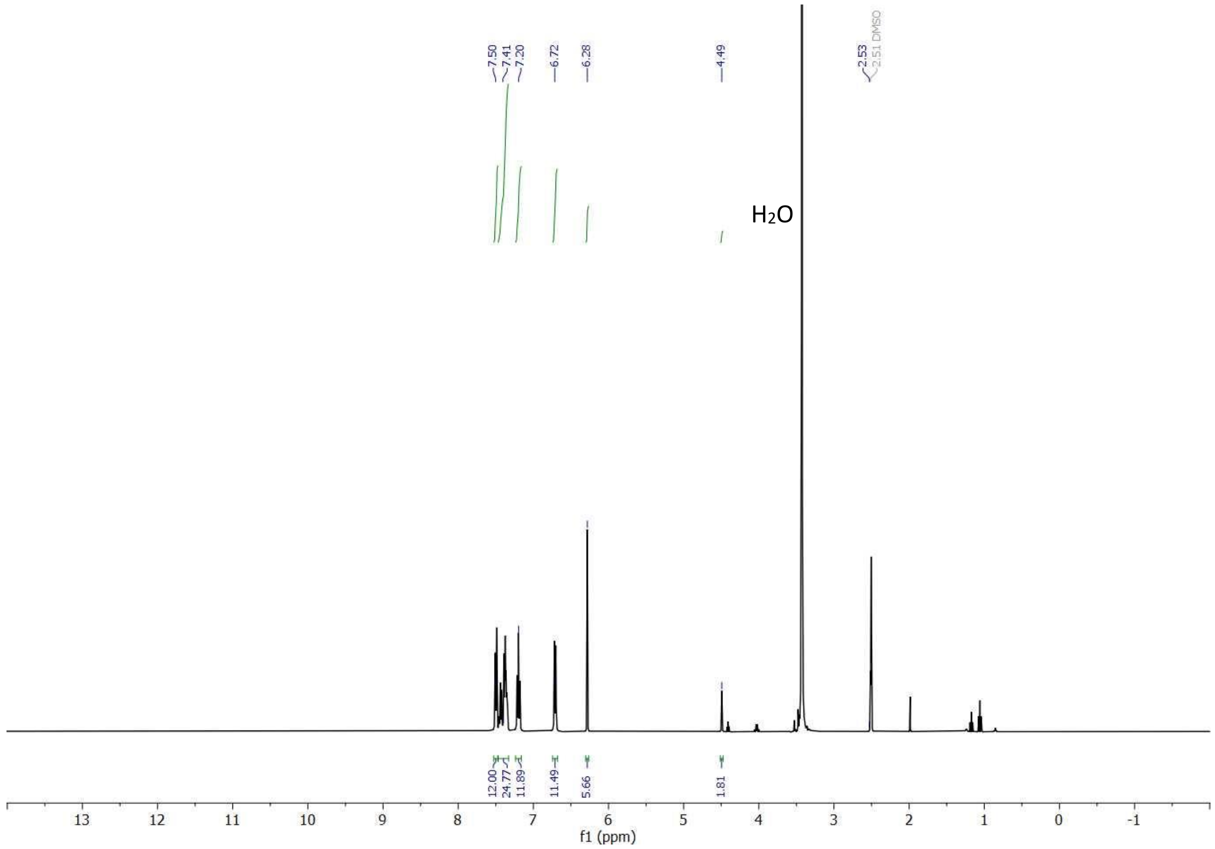

Figure S3: ^{1}H NMR spectrum (DMSO-d_6, 400 MHz) of compound **2**.

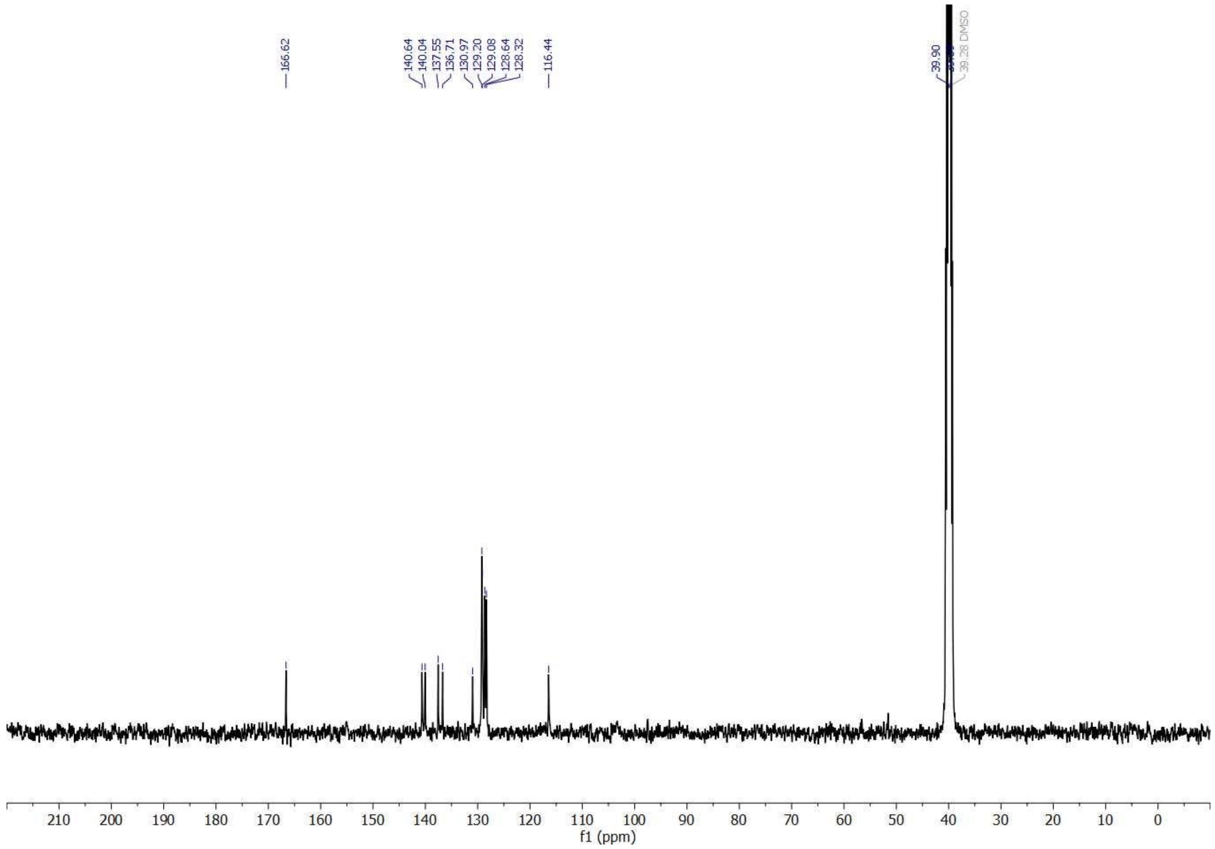

Figure S4: ^{13}C NMR spectrum (DMSO-d_6, 100 MHz) of compound **2**.

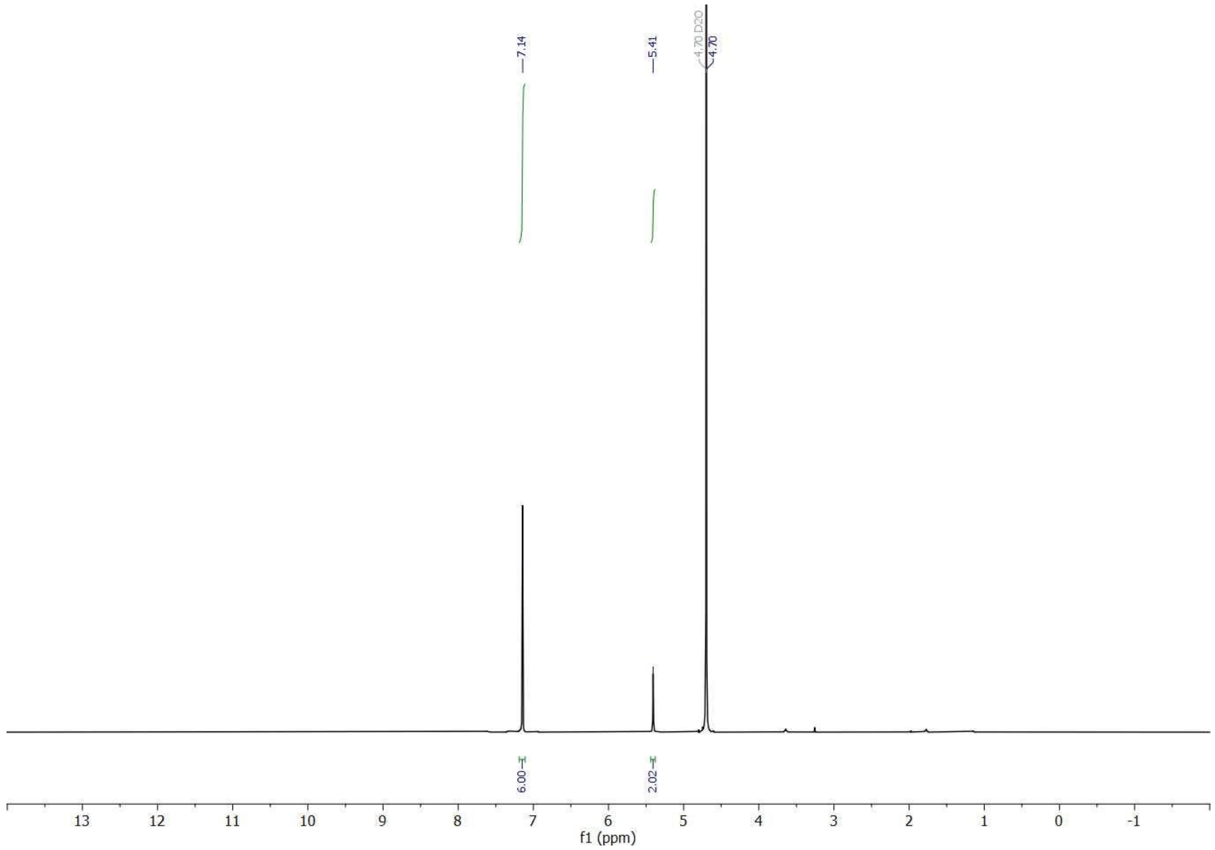

Figure S5: ^{1}H NMR spectrum (D_2O, 400 MHz) of compound **3**.

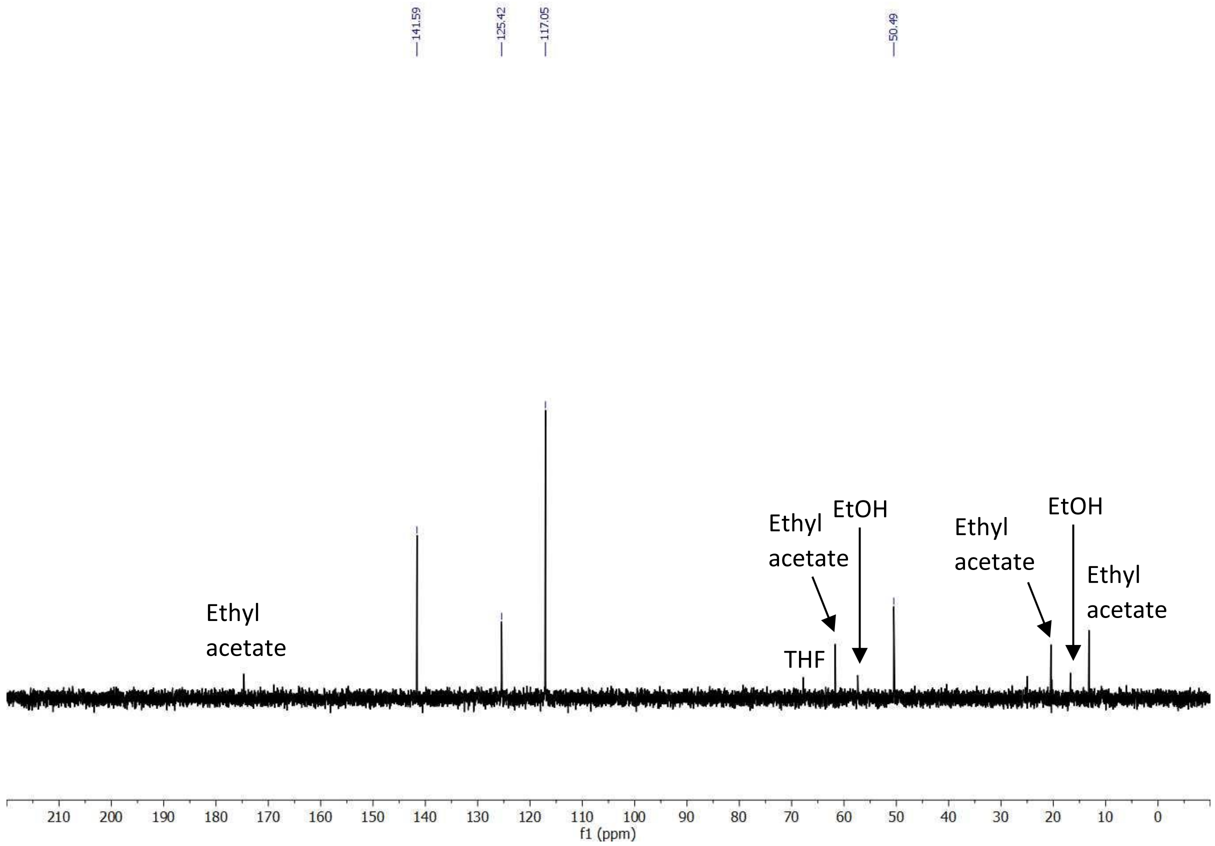

Figure S6: ^{13}C NMR spectrum (D_2O, 100 MHz) of compound **3**.

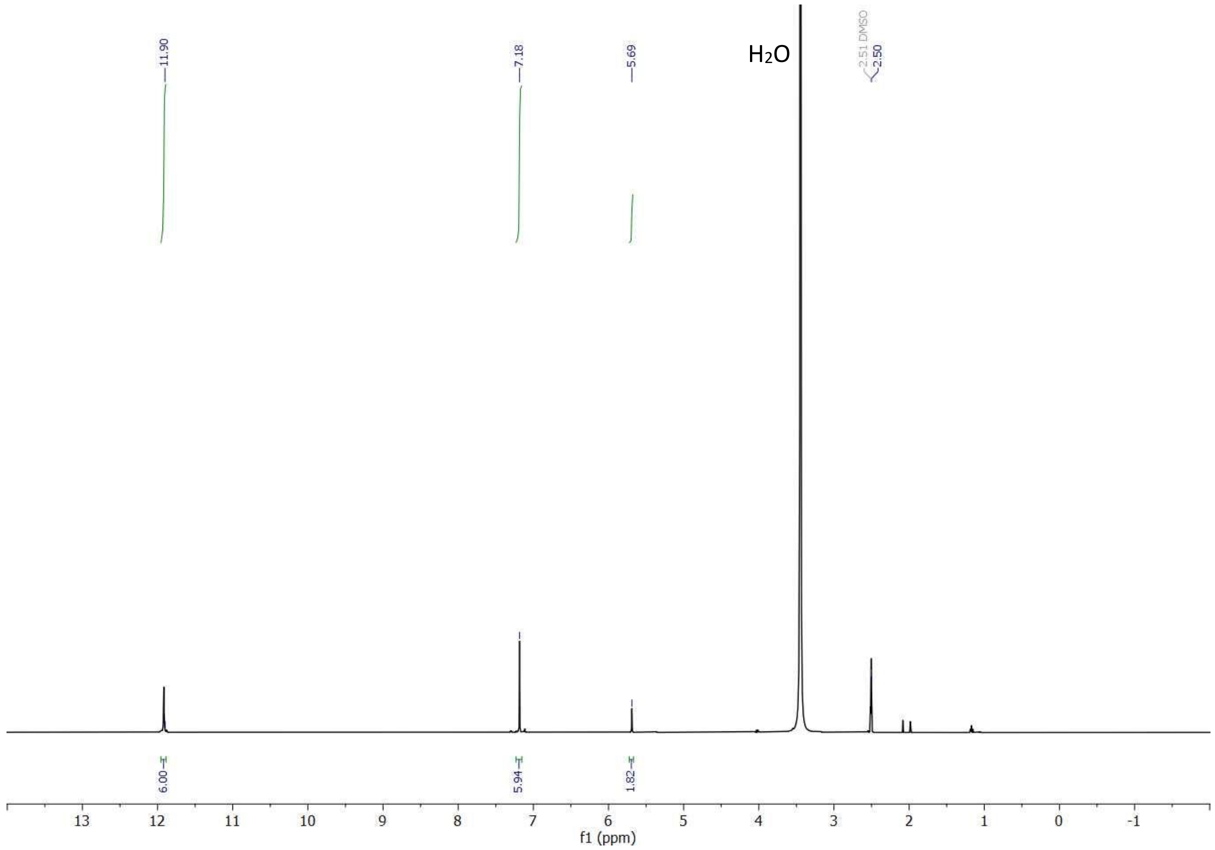

Figure S7: ^{1}H NMR spectrum (DMSO-d_6, 400 MHz) of TH5.

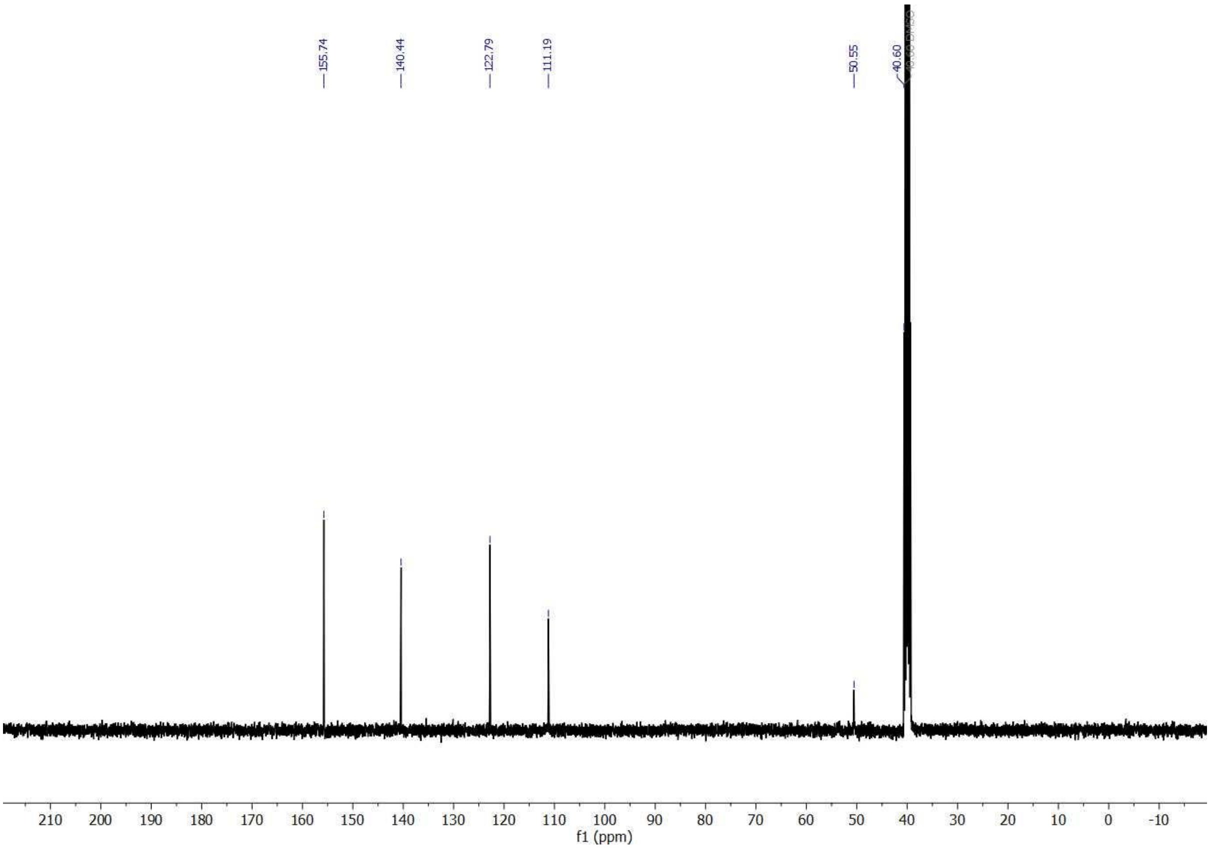

Figure S8: ^{13}C NMR spectrum (DMSO-d_6, 100 MHz) of TH5.

Table S3: SCXRD data for TH5 crystals analyzed from crystallization screen.

Name	Crystallization solvents	Space group	Lattice	a (Å)	b (Å)	c (Å)	α (°)	β (°)	γ (°)
TH5-DMF-THF	DMF/THF	*Pbca*	oP	32.30	15.42	38.44	90	90	90
TH5-NMP	NMP	$C222_1$	oC	13.54	20.10	16.24	90	90	90
TH5α-DMF-$CHCl_3$	DMF/$CHCl_3$	$P6_3/mmc$	hP	21.17	21.17	10.97	90	90	120
TH5α-acetone	DMF/$CHCl_3$ (acetone-exchanged)	$P6_3/mmc$	hP	21.36	21.36	11.03	90	90	120
TH5-DMF-1,4-dioxane	DMF/1,4-dioxane	$P6_3/mmc$	hP	21.38	21.38	11.19	90	90	120
TH5-DMF-acetone	DMF/acetone	$C222_1$	oC	13.07	19.65	16.66	90	90	90
TH5-DMF-EA	DMF/Ethyl acetate	*Pbca*	oP	33.70	15.89	39.59	90	90	90
TH5-DMSO-EtOH	DMSO/EtOH	$C222_1$	oC	13.05	19.11	16.45	90	90	90
TH5-DMF-IPA	DMF/IPA	*Pbca*	oP	32.74	15.84	39.46	90	90	90

Crystal data for TH5-DMF-THF (CCDC deposition number: 2241789). Formula: $2(C_{26}H_{14}N_6O_6)$, $2.75(C_3H_7NO)$; M = 1213.88 g mol^{-1}, orthorhombic *Pbca*, yellow block-shaped crystals; a = 32.3002(18) Å, b = 15.4229(6) Å, c = 38.436(2) Å, V = 19147.3(17) Å^3; ρ = 0.842 g cm^{-3}; μ(Mo-Kα) = 0.062 mm^{-3}; F(000) = 5040; crystal size = 0.39 × 0.22 × 0.11 mm; T = 100 K; 91425 reflections measured, 9965 unique (R_{int} = 0.0752), 6877 observed ($I > 2\sigma(I)$); R_1 = 0.0936 for observed and R_1 = 0.1190 for all reflections; wR_2 = 0.2999 for all reflections; max/min difference electron density = 0.387 and -0.287 e·Å^{-3}; parameters/reflections/restraints = 819/9965/97; GOF = 1.067.

Crystal data for TH5α-acetone (CCDC deposition number: 2241788). Formula: $C_{26}H_{14}N_6O_6$, $(C_3H_6O)_n$; M = 506.43 g mol^{-1}, hexagonal $P6_3/mmc$, orange block-shaped crystal; a = 21.3790(11) Å, c = 11.0395(4) Å, V = 4369.7(5) Å^3; ρ = 0.385 g cm^{-3}; μ(Mo-Kα) = 0.028 mm^{-3}; F (000) = 520; crystal size = 0.3 × 0.3 × 0.1 mm; T = 200 K; 45552 reflections measured, 1222 unique (R_{int} = 0.0525), 1042 observed ($I > 2\sigma(I)$); R_1 = 0.0572 for observed and R_1 = 0.0728 for all reflections; wR_2 = 0.1766 for all reflections; max/min difference electron density = 0.122 and -0.168 e·Å^{-3}; parameters/reflections/restraints = 40/1221/0; GOF = 1.169. The highest q-peak in the large solvent-accessible voids was 1, and it was not feasible to accurately model the disordered electron density. Therefore, we used the SQUEEZE routine in Platon during the final refinement cycles, which reduced the R_1(obs) value from ~21% to ~ 6%. SQUEEZE removed 579 electrons from an interconnected 3538 Å^3 void.

Crystal data for TH5-DMSO-EtOH (CCDC deposition number: 2241787). Formula: $C_{26}H_{14}N_6O_6$, $4.5(C_2H_6OS)$, H_2O; M = 874.01 g mol^{-1}, orthorhombic $C222_1$, yellow block-shaped crystals; a = 13.0635(2) Å, b = 19.1180(4) Å, c = 16.4710(3) Å, V = 4113.60(13) Å^3; Z = 4; ρ = 1.411 g cm^{-3}; μ(Mo-Kα) = 0.322 mm^{-3}; F(000) = 1828; crystal size = 0.29 × 0.24 × 0.20 mm; T = 100 K; 19221 reflections measured, 5527 unique (R_{int} = 0.015), 5343 for observed ($I > 2\sigma(I)$); R_1 = 0.0586 for observed and R_1 = 0.0598 for all reflections; wR_2 = 0.1732 for all reflections; max/min difference electron density = 0.626 and -0.462 e·Å^{-3}; parameters/reflections/restraints = 401/5527/89; GOF = 1.004.

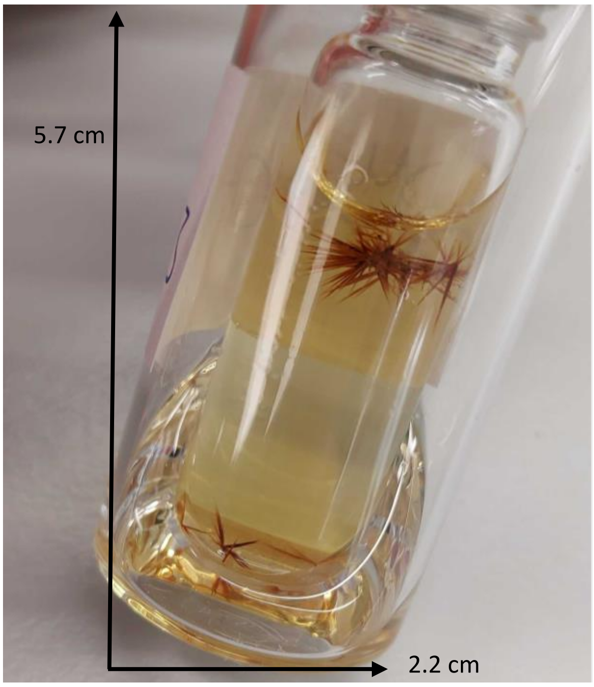

Figure S9: Crystals of TH5α-DMF-$CHCl_3$. The dimensions of the larger vial are shown for scale.

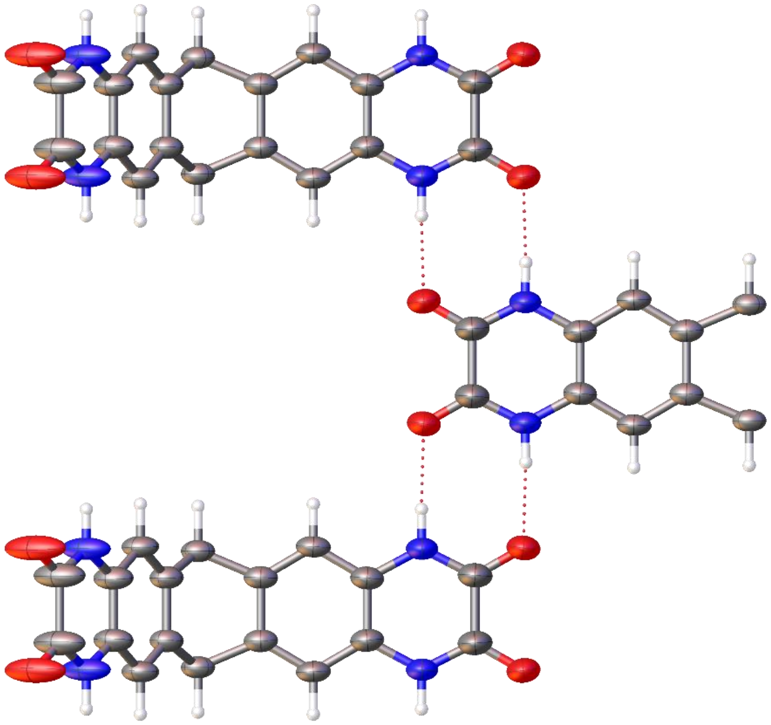

Figure S10: N-H···O hydrogen bonding between neighbouring TH5 molecules in the TH5α-DMF-$CHCl_3$ crystal structure. Ellipsoids are displayed using a 50% probability level.

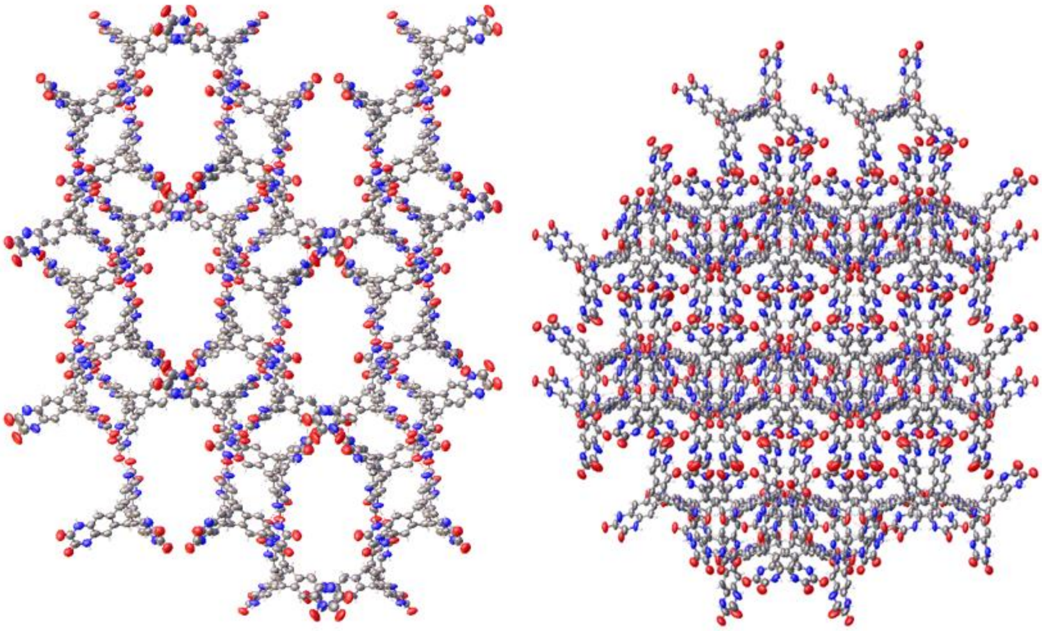

Figure S11: Displacement ellipsoid plot from the single crystal structure of the TH5-DMF-THF, shown along crystallographic *a* (left) and *c* (right) axes. The crystal structure is comparable to that of TH5-DMF-EA and TH5-DMF-IPA. Ellipsoids are displayed at a 50% probability level.

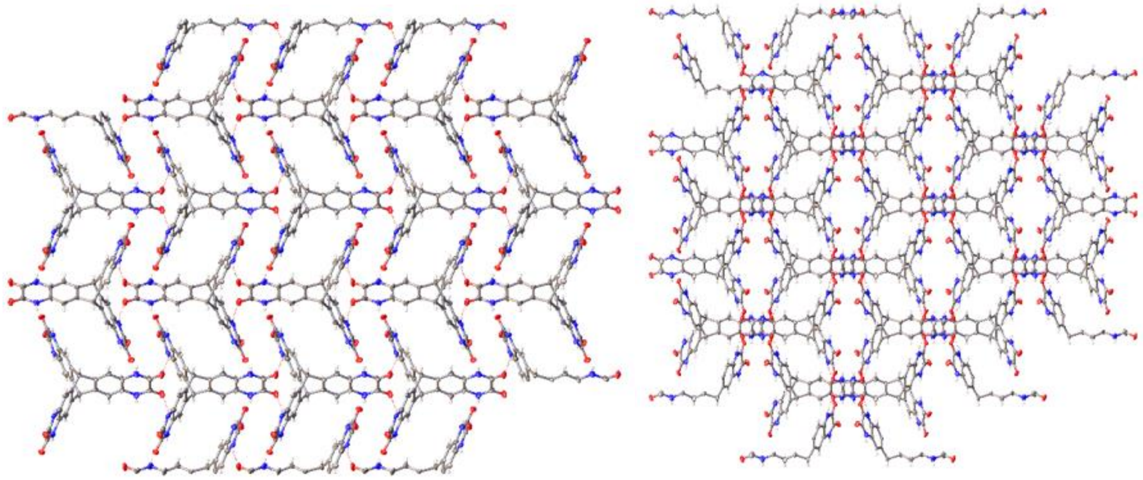

Figure S12: Displacement ellipsoid plot from the single crystal structure of TH5-DMSO-EtOH, shown along crystallographic *a* (left) and *c* (right) axes. The crystal structure is comparable to that of the TH5-DMF-acetone and TH5-NMP solvates. Ellipsoids are displayed at a 50% probability level.

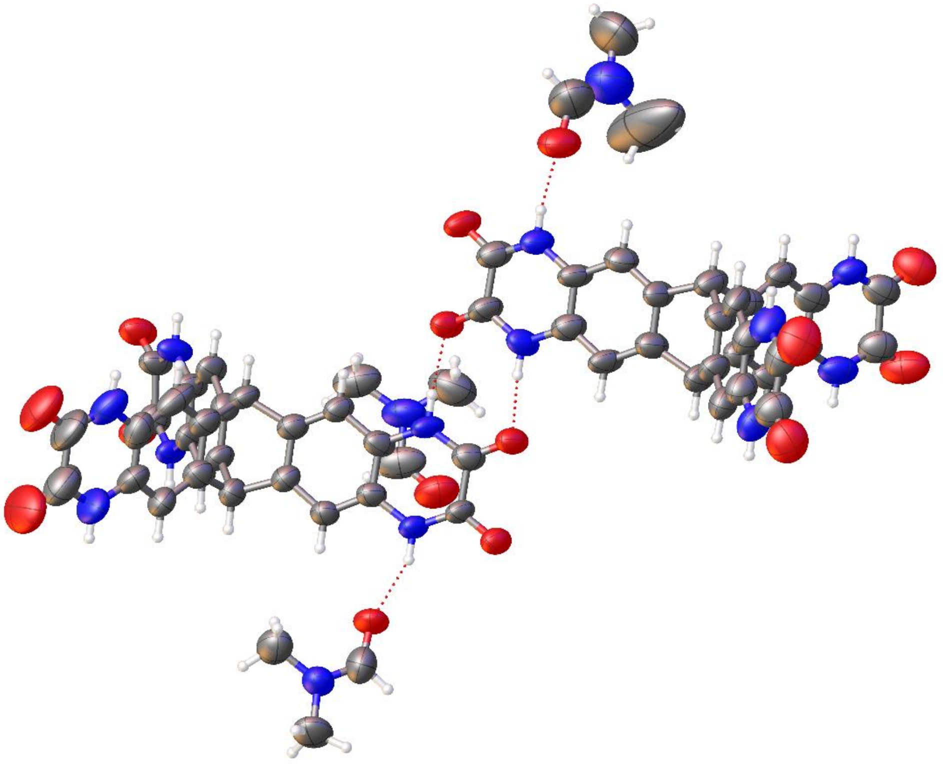

Figure S13: Displacement ellipsoid plot from the single crystal structure of the TH5-DMF-THF, showing the hydrogen-bonding interaction of TH5 with DMF. Ellipsoids are displayed at a 50% probability level.

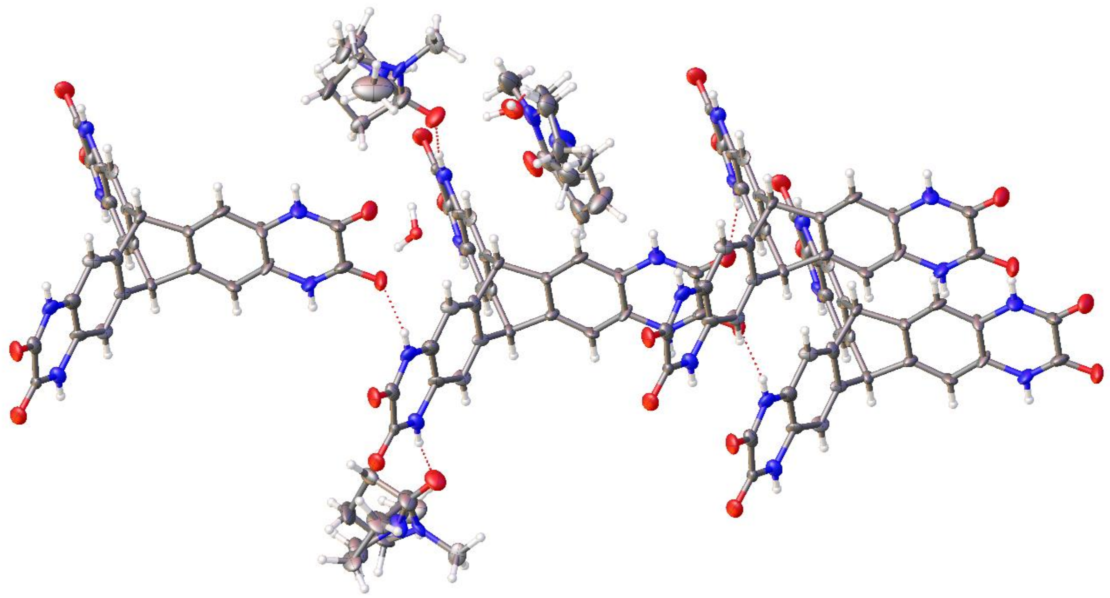

Figure S14: Displacement ellipsoid plot from the single crystal structure of the NMP solvate of TH5-NMP, showing the hydrogen-bonding interaction of TH5 with NMP. Ellipsoids are displayed at a 50% probability level.

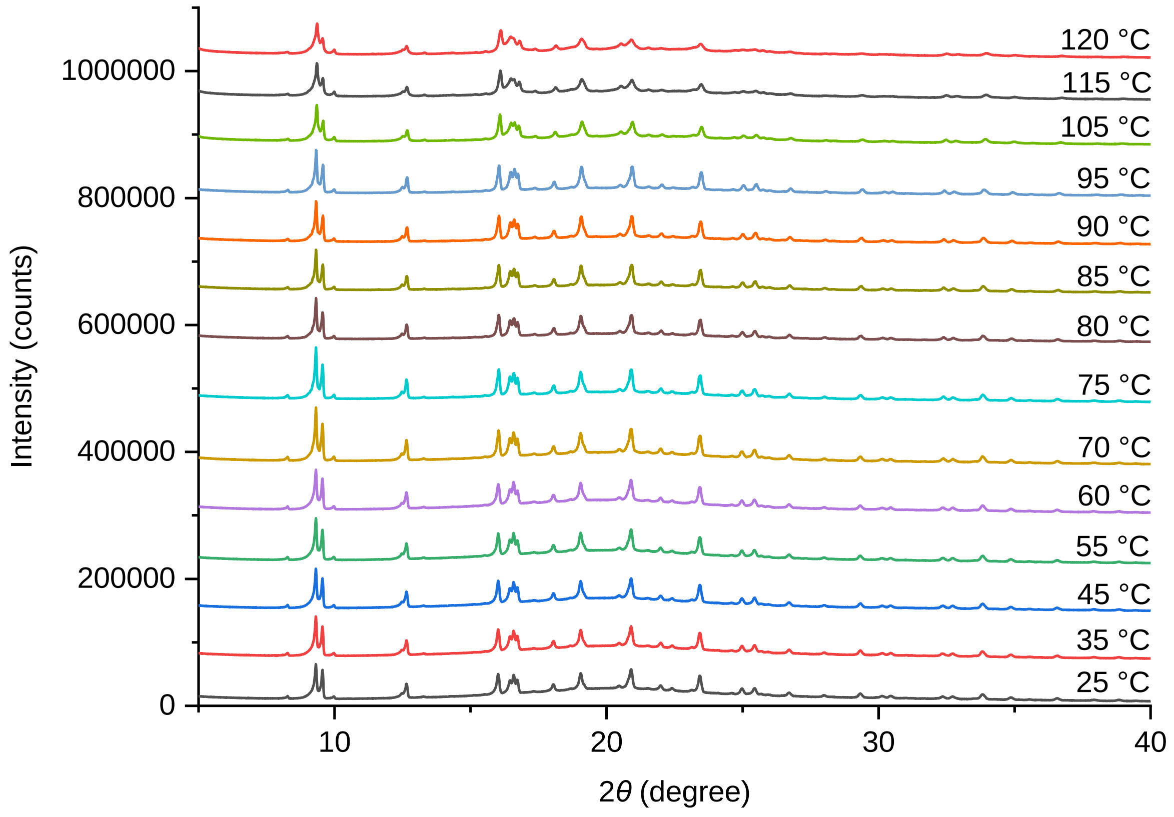

Figure S15: Variable-temperature PXRD study of TH5α-DMF-$CHCl_3$.

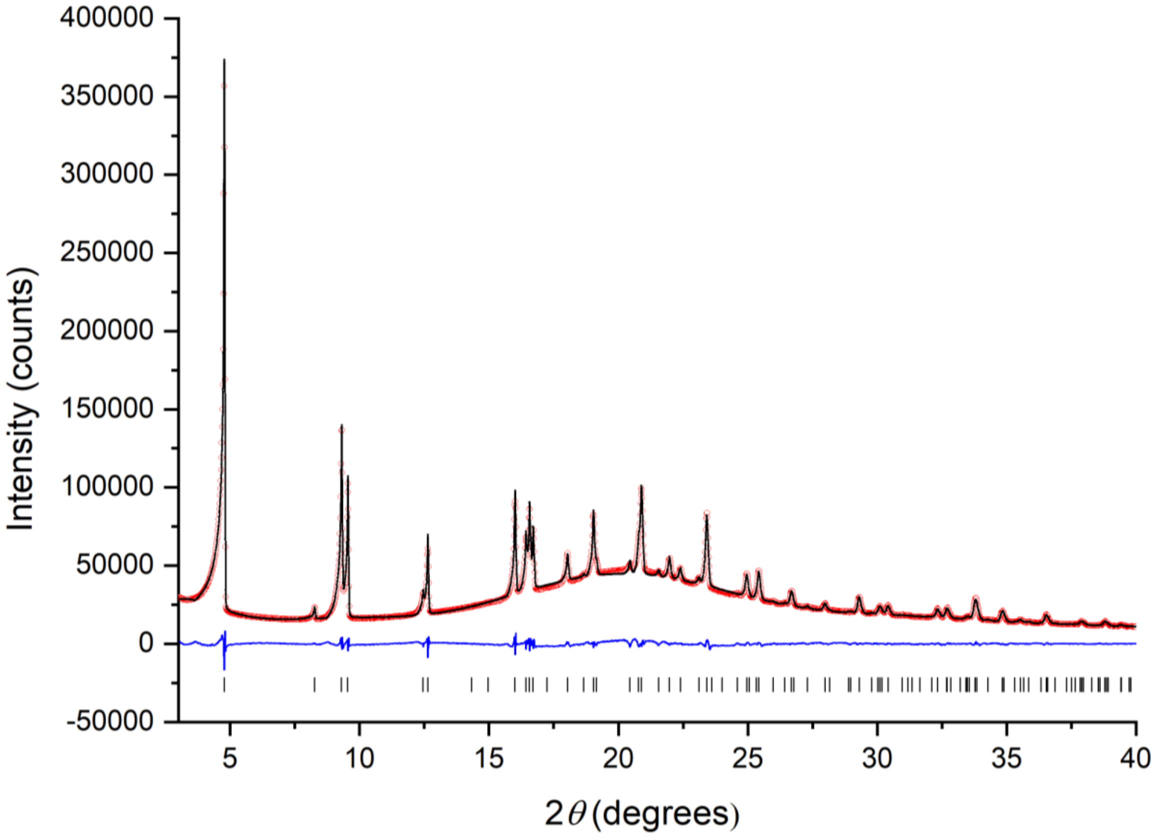

Figure S16: PXRD pattern fitting of TH5α-acetone with Pawley refinement (Cu-Kα). The PXRD pattern was recorded in a capillary in acetone solvent at 25 °C, and the capillary was spun during the measurement to improve averaging. Red circles: experimental PXRD pattern, black line: fitting pattern, blue curve: difference between experimental and refinement, black bars: reflection positions. R_p = 2.065%, R_{wp} = 2.615% ($P6_3/mmc$, $a = b$ = 21.394 Å, c = 11.068 Å, V = 4387.2 Å^3).

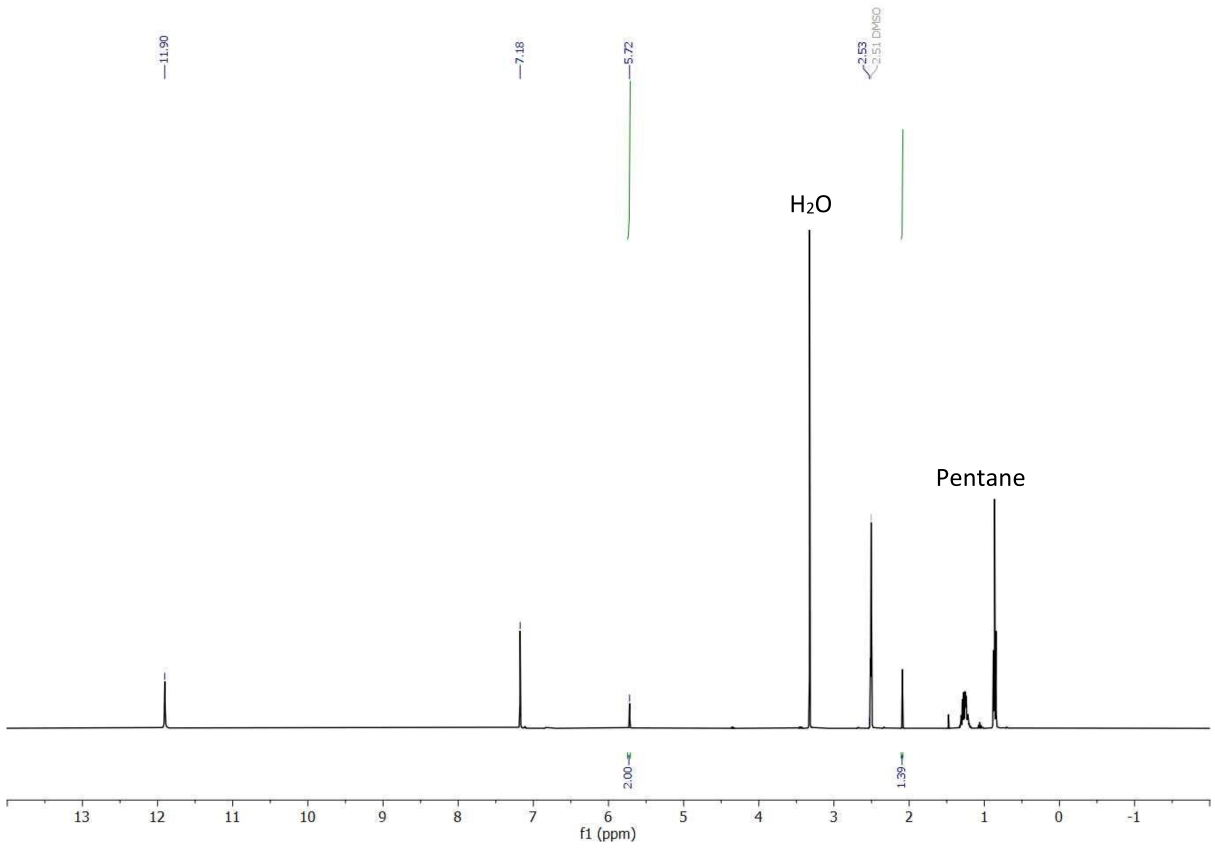

Figure S17: ^{1}H NMR spectrum (DMSO-d_6, 400 MHz) of TH5α after exchange with pentane (TH5α-pentane). The singlet at δ 2.09 ppm is residual acetone solvent that appeared to remain in the crystal pores.

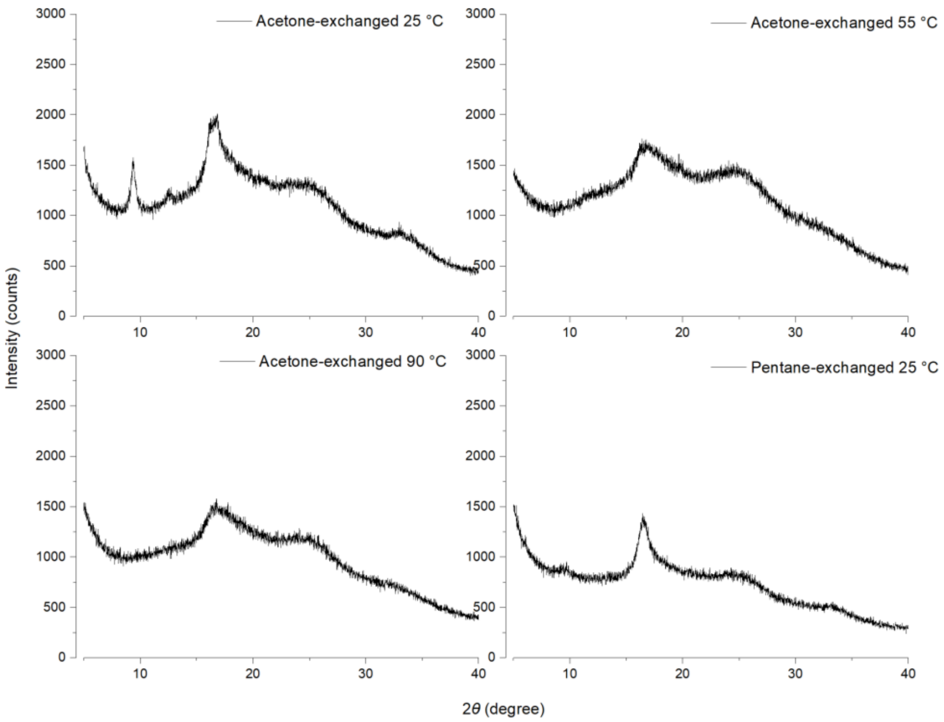

Figure S18: PXRD patterns for TH5α-acetone and TH5α-pentane after heat/vacuum activation.

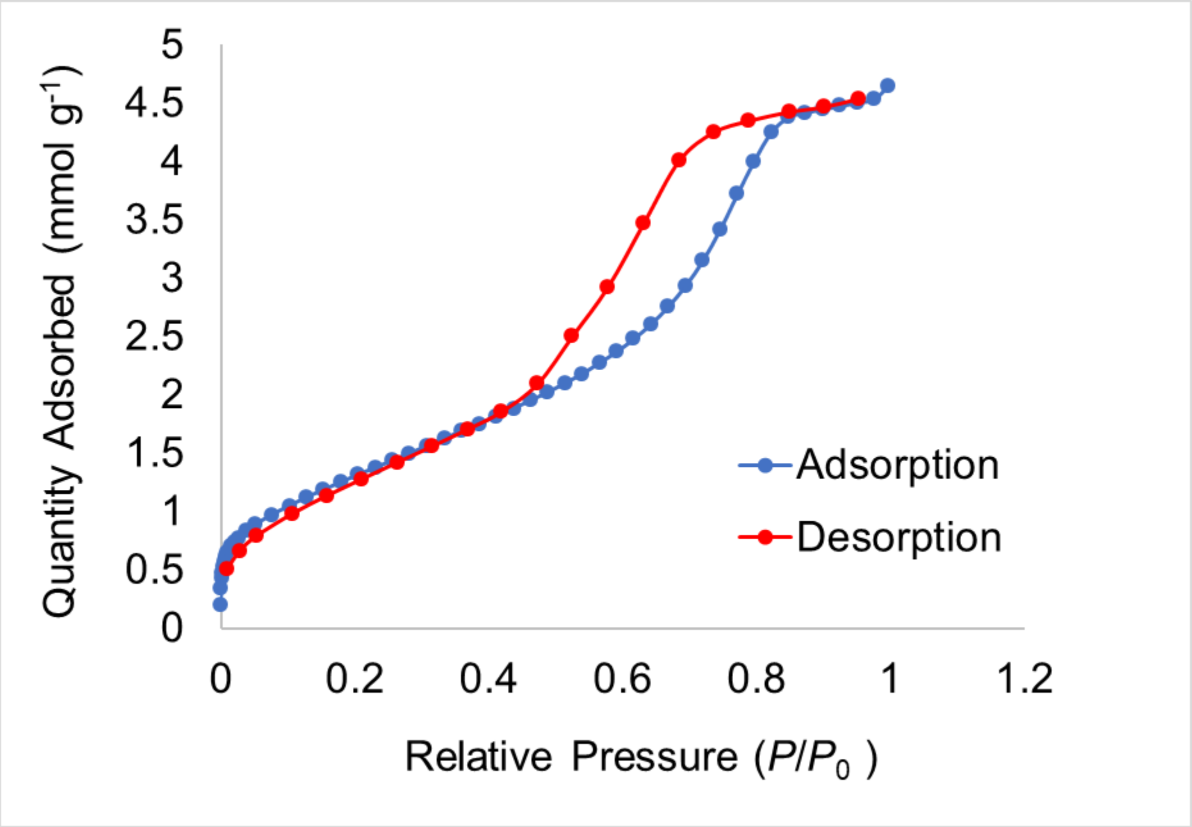

Figure S19: N_2 sorption isotherm at 77 K for TH5α-acetone material activated at 25 °C under vacuum. Crystals were degassed on port at 25 °C.

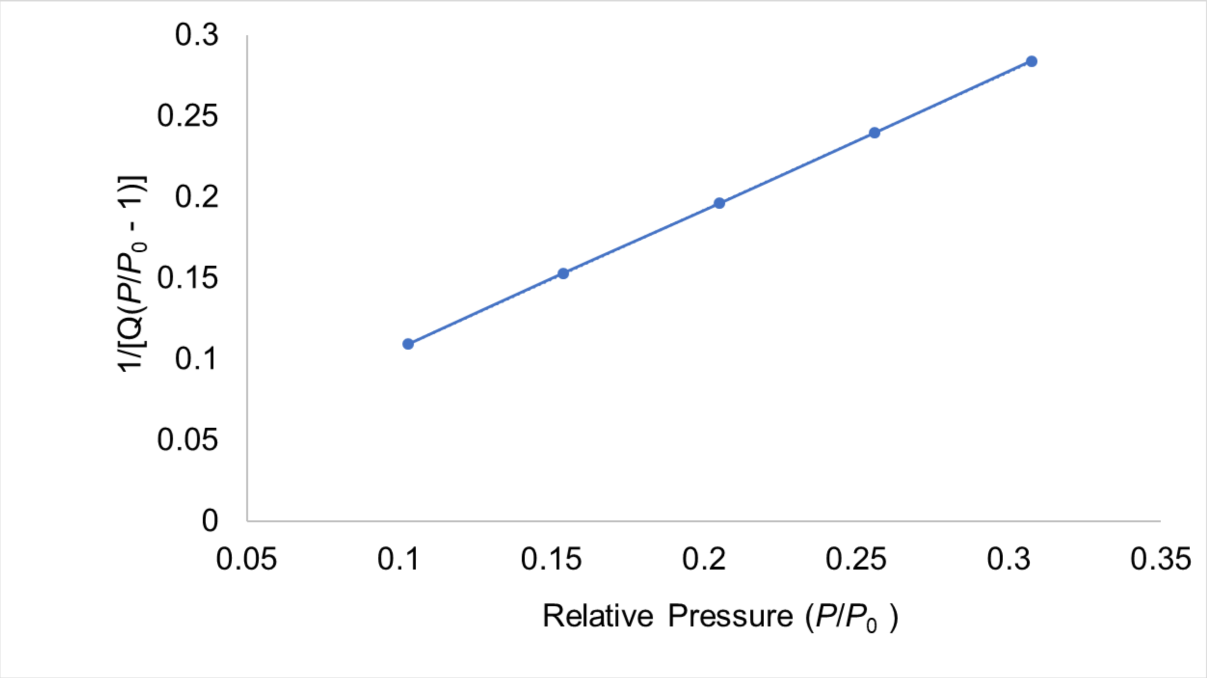

Figure S20: BET surface area plot from TH5α-acetone material activated at 25 °C under vacuum, calculated from N_2 isotherm at 77 K. BET surface area 111.6 ± 0.3 m^2 g^{-1}; Intercept 0.021432 ± 0.000519 g $mmol^{-1}$; C 40.803537; Qm 1.14352 mmol g^{-1}; correlation coefficient 0.9999883.

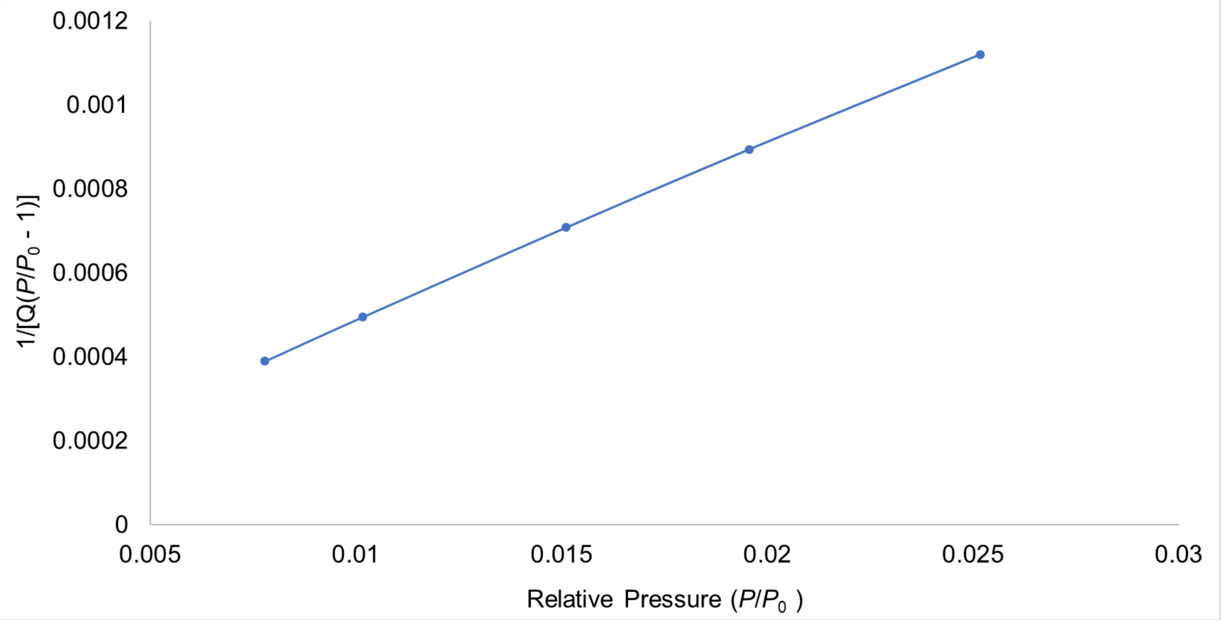

Figure S21: BET surface area plot from TH5α-scCO_2-activated material, calculated from N_2 isotherm at 77 K. BET surface area 3,284.4 ± 38.9 m^2 g^{-1}; Intercept 0.000076 ± 0.000008 g $mmol^{-1}$; C 388.357639; Qm 33.66563 mmol g^{-1}; correlation coefficient 0.9997891.

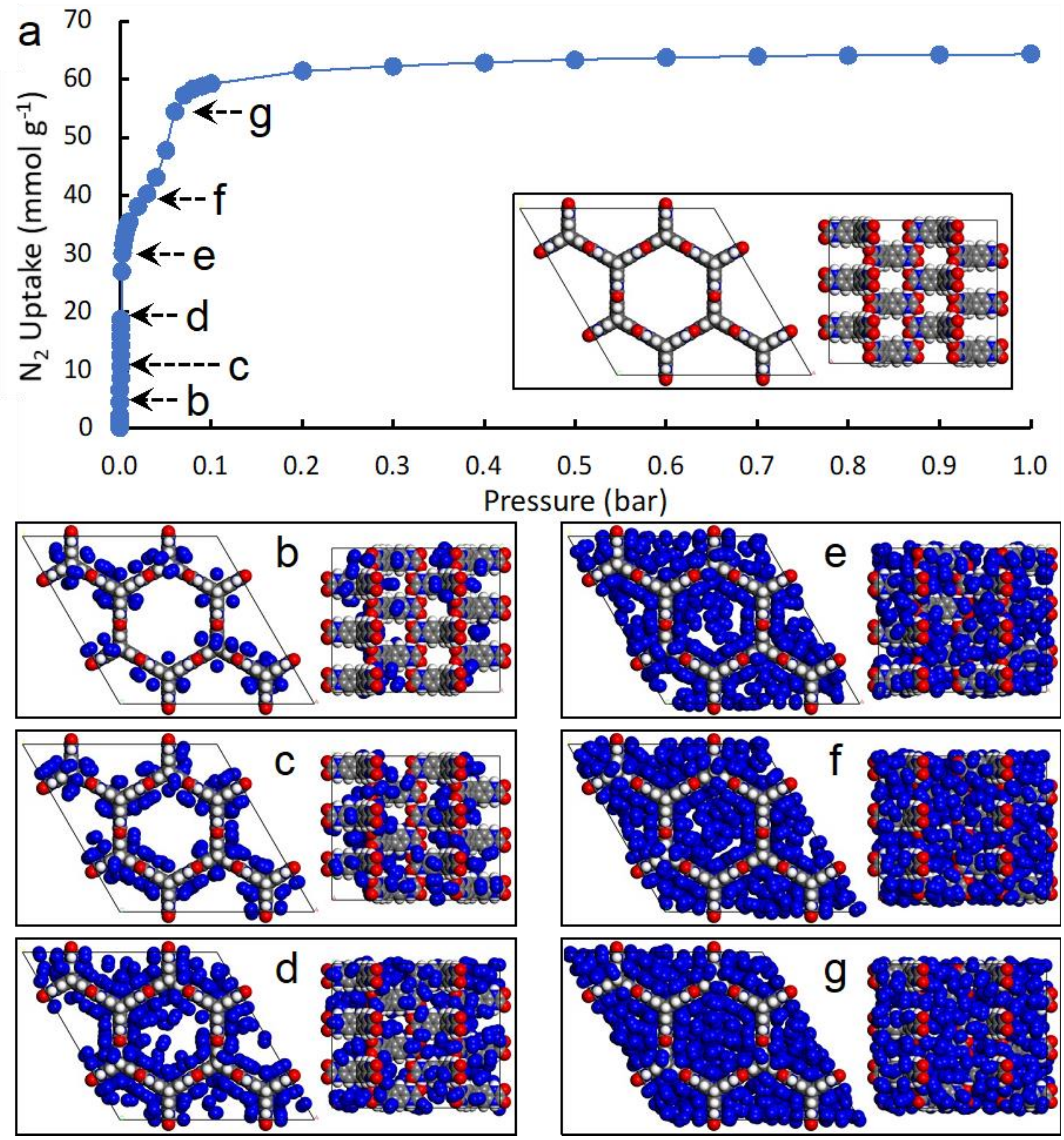

Figure S22: Predicted N_2 adsorption isotherm for TH5-A, (a). (b–g) Snapshots from the adsorption simulations showing sequential pore filling corresponding to the points labelled in (a).

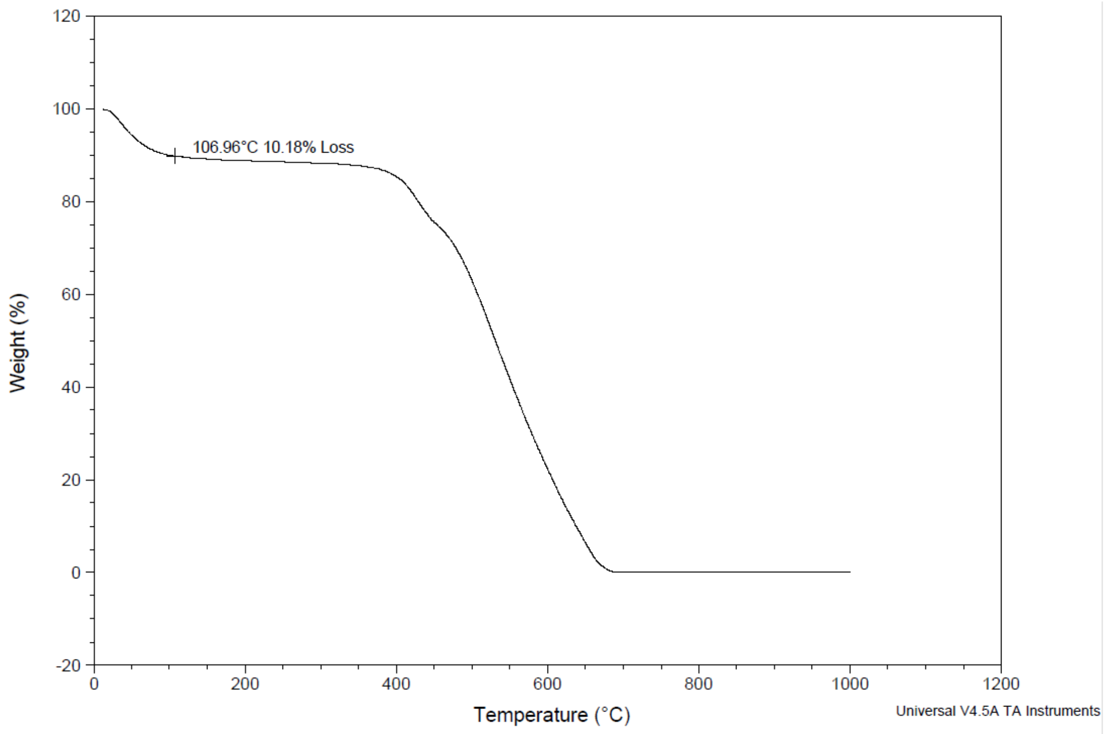

Figure S23: TGA plot of TH5α-scCO_2-activated recorded after gas sorption. Degas temperature 25 °C. The plot shows 10.18% weight loss at 106.96 °C, corresponding to acetone solvent remaining in the crystal pores.

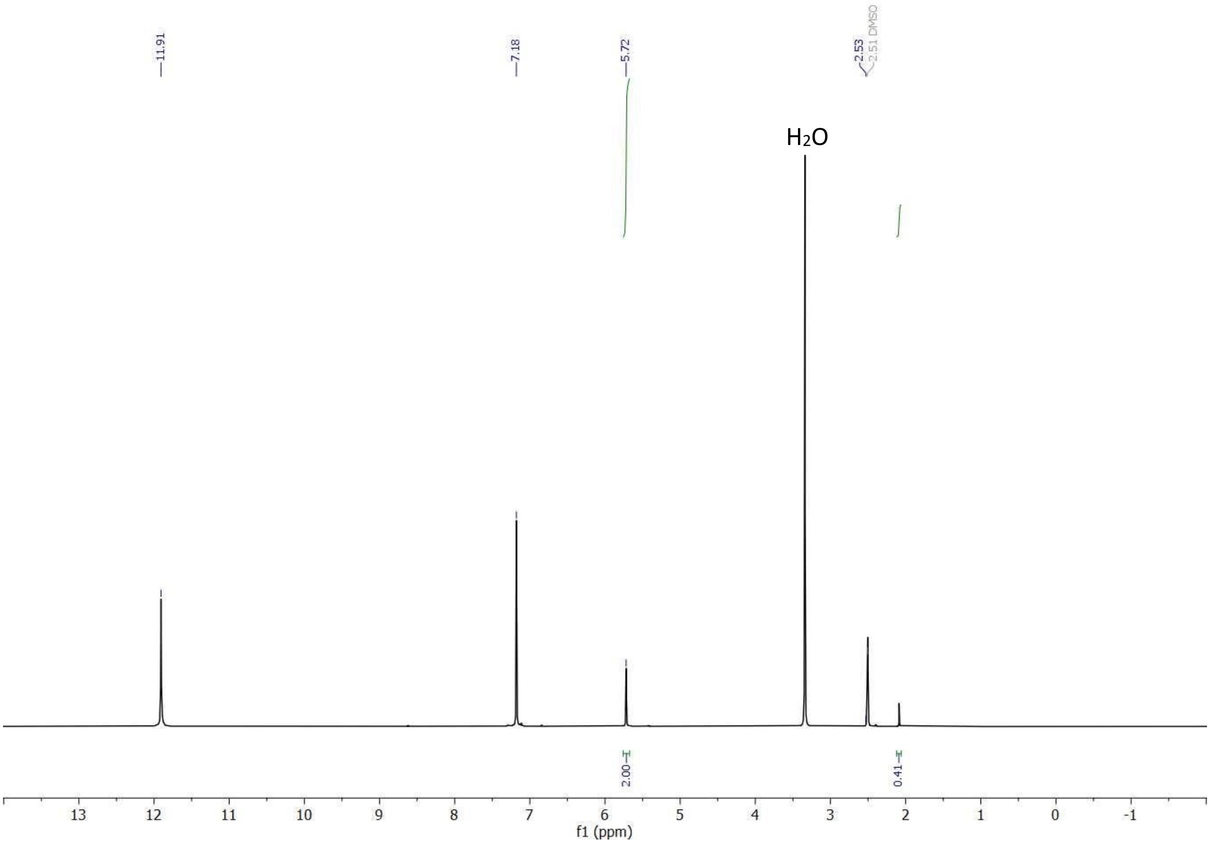

Figure S24: ^{1}H NMR spectrum (DMSO-d_6, 400 MHz) of TH5α-scCO_2-activated recorded after gas sorption. Degas temperature 25 °C. The singlet peak at δ 2.09 ppm is retained acetone solvent that appeared to remain in the crystal pores.

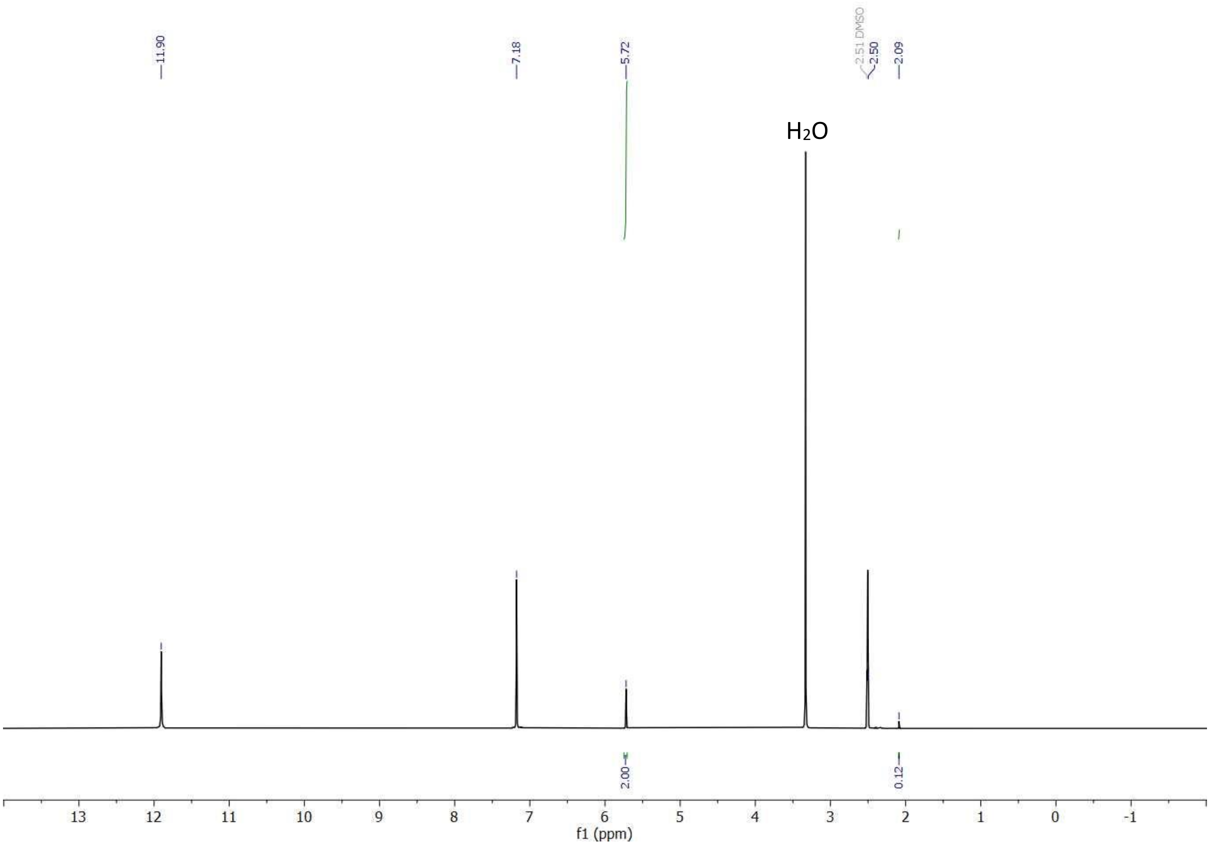

Figure S25: ^{1}H NMR spectrum (DMSO-*d*6, 400 MHz) of TH5α-scCO2-activated after gas sorption. Degas temperature 40 °C. The singlet peak at δ 2.09 ppm is retained acetone solvent that appeared to remain in crystal pores.

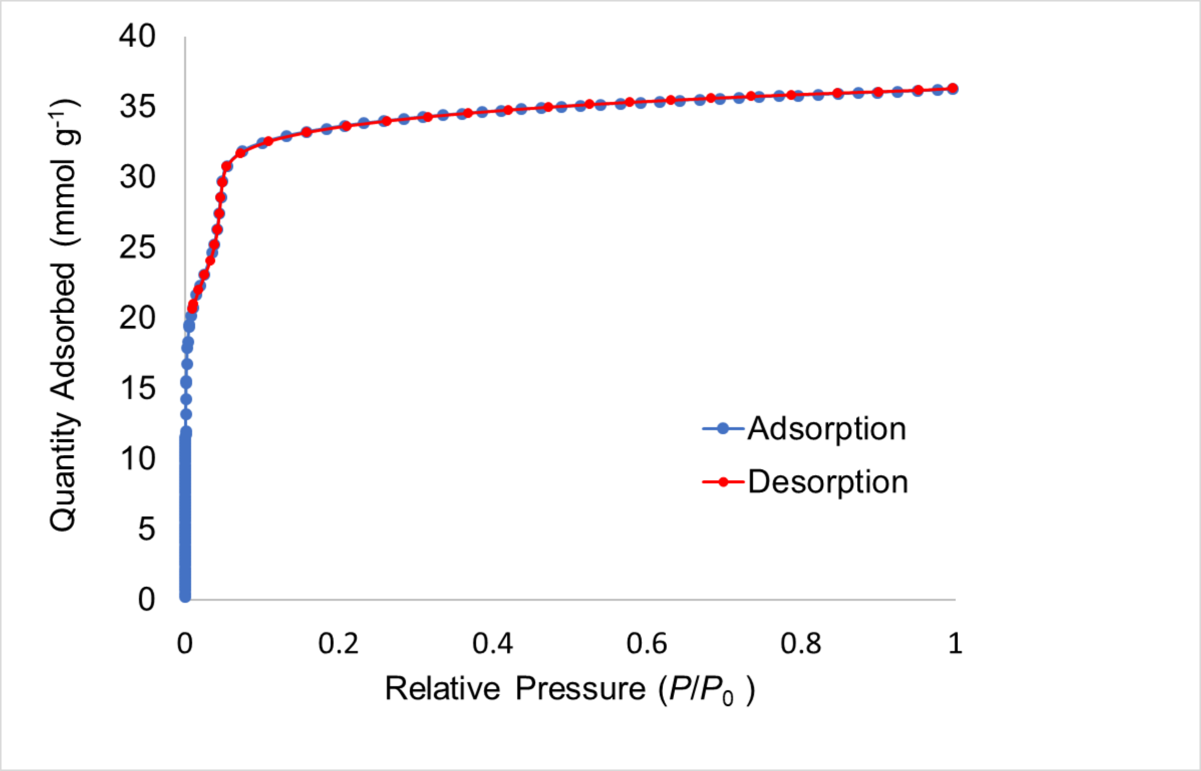

Figure S26: N_2 sorption isotherm at 77 K for TH5α-scCO2-activated material recorded after degassing at 40 °C.

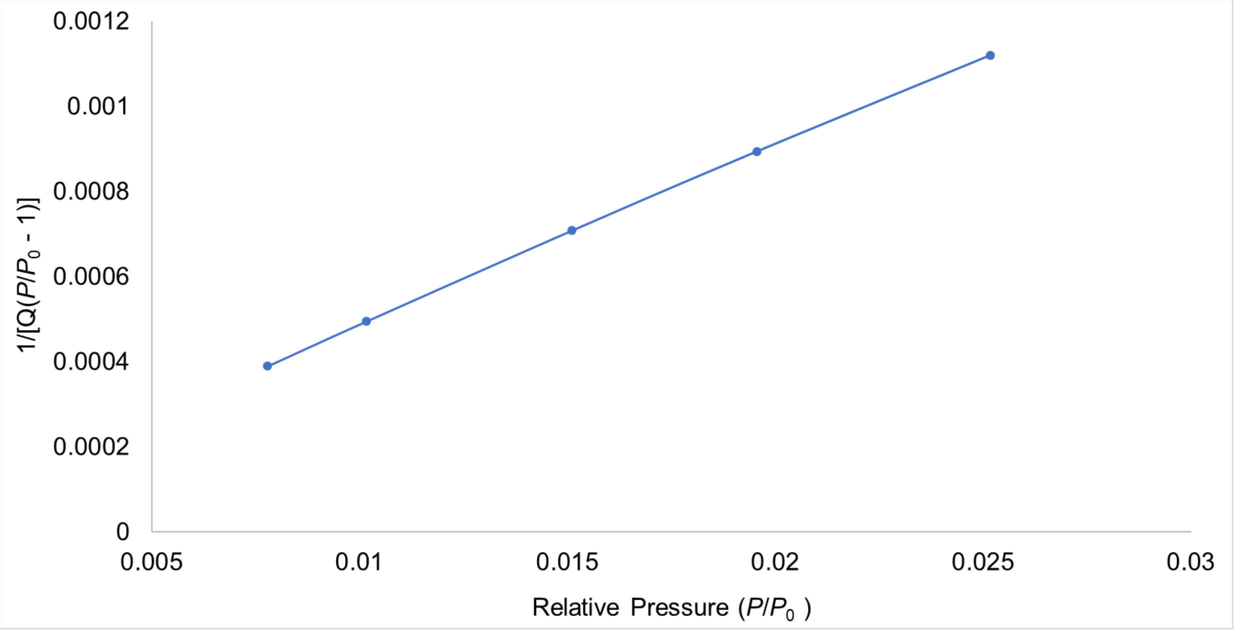

Figure S27: BET surface area plot for TH5α-scCO_2-activated material recorded after degassing at 40 °C, calculated from N_2 isotherm at 77 K. BET surface area 2,315.6 ± 23.8 m² g^{-1}; intercept 0.000067 ± 0.000007 g mmol^{-1}; C 626.614396; Qm 23.73580 mmol g^{-1}; correlation coefficient 0.9998407.

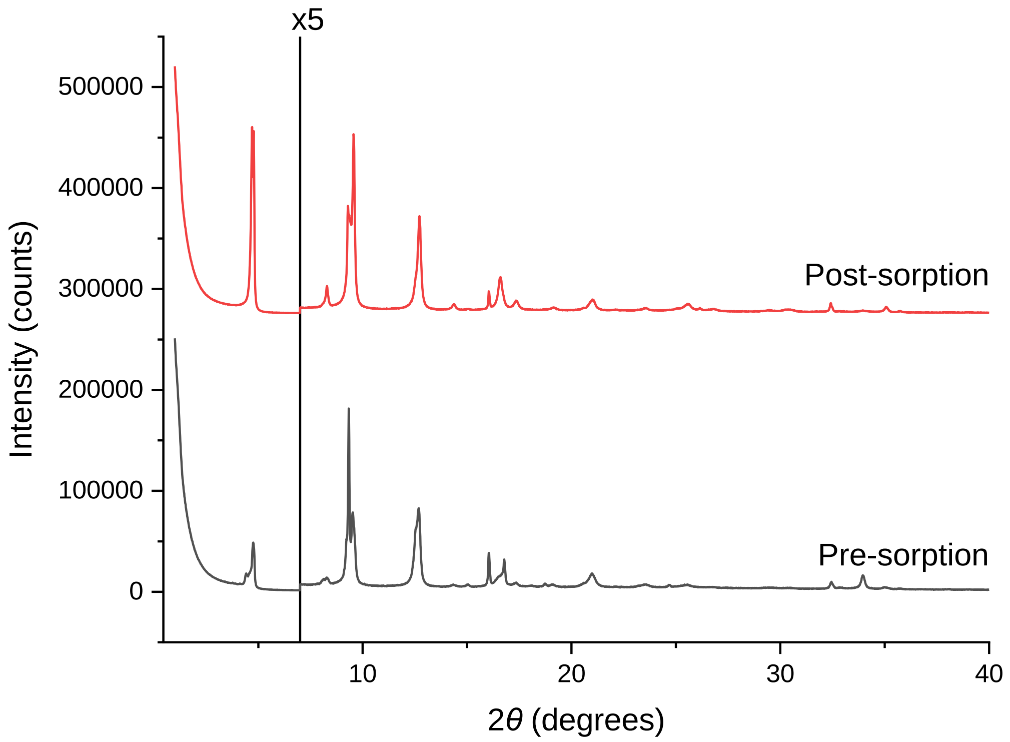

Figure S28: Overlay of PXRD patterns of TH5α-scCO_2-activated materials recorded before (black) and after (red) gas sorption analysis with on-port degas at 40 °C. Intensities (counts) are multiplied x5 from $2\vartheta = 7$ (right of the vertical line).

4.0 References

[1] G. Sheldrick, *Acta Cryst.* **2015**, *A71*, 3–8.

[2] G. Sheldrick, *Acta Cryst.* **2015**, *C71*, 3–8.

[3] L. J. B. O. V. Dolomanov R. J. Gildea, J. A. K. Howard and H. Puschmann, *J. Appl. Cryst.* **2009**, *42*, 339–341.

[4] A. L. Spek, *Acta Cryst.* **2015**, *C71*, 9–18.

[5] A. L. Spek, *J. Appl. Crystallogr.* **2003**, *36*, 7–13.

[6] J. Rouquerol, F. Rouquerol, C. Pérès, Y. Grillet, M. Boudellal, in *Characterisation of Porous Solids* (Eds.: S.J. Gregg, K.S.W. Sing, H.F. Stoeckli), Society Of Chemical Industry, London, **1979**, p. 107.

[7] C. L. Hilton, C. R. Jamison, H. K. Zane, B. T. King, *J. Org. Chem.* **2009**, *74*, 405–407.

[8] M. G. Rabbani, T. E. Reich, R. M. Kassab, K. T. Jackson, H. M. El-Kaderi, *Chem. Commun.* **2012**, *48*, 1141–1143.

[9] M. Mastalerz, S. Sieste, M. Cenić, I. M. Oppel, *J. Org. Chem.* **2011**, *76*, 6389–6393.

[10] C. Zhao, L. Chen, Y. Che, Z. Pang, X. Wu, Y. Lu, H. Liu, G. M. Day, A. I. Cooper, *Nat. Commun.* **2021**, *12*, 817.

[11] D. H. Case, J. E. Campbell, P. J. Bygrave, G. M. Day, *J. Chem. Theory Comput.* **2016**, *12*, 910–924.

[12] S. L. Price, M. Leslie, G. W. A. A. Welch, M. Habgood, L. S. Price, P. G. Karamertzanis, G. M. Day, *Phys. Chem. Chem. Phys.* **2010**, *12*, 8478.

[13] J. A. Chisholm, S. Motherwell, *J. Appl. Crystallogr.* **2005**, *38*, 228–231.

[14] D. Dubbeldam, S. Calero, D. E. Ellis, R. Q. Snurr, *Mol. Simul.* **2016**, *42*, 81–101.

[15] S. L. Mayo, B. D. Olafson, W. A. Goddard, *J. Phys. Chem.* **1990**, *94*, 8897–8909.

[16] J. J. Potoff, J. I. Siepmann, *AIChE J.* **2001**, *47*, 1676–1682.

[17] Gaussian 09, Revision A.02, M. J. Frisch, G. W. Trucks, H. B. Schlegel, G. E. Scuseria, M. A. Robb, J. R. Cheeseman, G. Scalmani, V. Barone, G. A. Petersson, H. Nakatsuji, X. Li, M. Caricato, A. Marenich, J. Bloino, B. G. Janesko, R. Gomperts, B. Mennucci, H. P. Hratchian, J. V. Ortiz, A. F. Izmaylov, J. L. Sonnenberg, D. Williams-Young, F. Ding, F. Lipparini, F. Egidi, J. Goings, B. Peng, A. Petrone, T. Henderson, D. Ranasinghe, V. G. Zakrzewski, J. Gao, N. Rega, G. Zheng, W. Liang, M. Hada, M. Ehara, K. Toyota, R. Fukuda, J. Hasegawa, M. Ishida, T. Nakajima, Y. Honda, O. Kitao, H. Nakai, T. Vreven, K. Throssell, J. A. Montgomery, Jr., J. E. Peralta, F. Ogliaro, M. Bearpark, J. J. Heyd, E. Brothers, K. N. Kudin, V. N. Staroverov, T. Keith, R. Kobayashi, J. Normand, K. Raghavachari, A. Rendell, J. C. Burant, S. S. Iyengar, J. Tomasi, M. Cossi, J. M. Millam, M. Klene, C. Adamo, R. Cammi, J. W. Ochterski, R. L. Martin, K. Morokuma, O. Farkas, J. B. Foresman, and D. J. Fox, Gaussian, Inc., Wallingford CT, **2016**.
